# Radiocesium in the Taiwan Strait and the Kuroshio east of Taiwan from 2018 to 2019

**DOI:** 10.1038/s41598-021-01895-y

**Published:** 2021-11-17

**Authors:** Wei-Jen Huang, Ming-Ta Lee, Kuei-Chen Huang, Kai-Jung Kao, Ming-An Lee, Yiing-Jang Yang, Sen Jan, Chen-Tung Arthur Chen

**Affiliations:** 1 Department of Oceanography, National Sun Yat-Sen University, Kaohsiung, Taiwan; 2 The Radiation Monitoring Center, Atomic Energy Council, Kaohsiung, Taiwan; 3 Department of Environmental Biology and Fisheries Science, National Taiwan Ocean University, Keelung, Taiwan; 4 Center of Excellence for Ocean Engineering, National Taiwan Ocean University, Keelung, 20224 Taiwan; 5 Institute of Oceanography, National Taiwan University, Taipei, Taiwan

**Keywords:** Ocean sciences, Marine chemistry

## Abstract

The release of anthropogenic radiocesium to the North Pacific Ocean (NPO) has occurred in the past 60 years. Factors controlling ^137^Cs (half-life, 30.2 year) and ^134^Cs (half-life, 2.06 year) activity concentrations in the Kuroshio east of Taiwan and the Taiwan Strait (latitude 20° N–27° N, longitude 116° E–123° E) remain unclear. This study collected seawater samples throughout this region and analyzed ^134^Cs and ^137^Cs activity concentrations between 2018 and 2019. A principal component analysis (PCA) was performed to analyze the controlling factors of radiocesium. Results of all ^134^Cs activity concentrations were below the detection limit (0.5 Bq m^−3^). Analyses of water column ^137^Cs profiles revealed a primary concentration peak (2.1–2.2 Bq m^−3^) at a depth range of 200–400 m (potential density σ_θ:_ 25.3 to 26.1 kg m^−3^). The PCA result suggests that this primary peak was related to density layers in the water column. A secondary ^137^Cs peak (1.90 Bq m^−3^) was observed in the near-surface waters (σ_θ_ = 18.8 to 21.4 kg m^−3^) and was possibly related to upwelling and river-to-sea mixing on the shelf. In the Taiwan Strait, ^137^Cs activity concentrations in the near-surface waters were higher in the summer than in the winter. We suggest that upwelling facilitates the vertical transport of ^137^Cs at the shelf break of the western NPO.

## Introduction

The global fallout from the atmospheric nuclear weapons tests during the late 1950s to early 1960s is the main source of artificial radiocesium to the world ocean^1,2^ and can still be discerned in the surface water, the subsurface, and deep waters (up to 600–1000 m) in the western North Pacific Ocean (NPO)^3^. After the Fukushima Daiichi nuclear power plant (FDNPP) accident on March 11th, 2011, additional anthropogenic radiocesium with long half-life ^137^Cs (30.2 year) and short half-life ^134^Cs (2.06 year) was released into the NPO^4–11^. Previous studies have estimated that the total amount of ^137^Cs released by the FDNPP accident ranged between 15 and 27 PBq, of which a very significant portion found its way to the atmosphere and ocean^5,12,13^. Traceable, FDNPP-derived ^137^Cs (latitude 37.42° N, longitude 141.03° E) follows the pathway of the North Pacific circulation toward the eastern NPO^6,9,14^. Thus 60 years after the first nuclear tests in the Pacific region, the fate of long-lives radionuclides on the western NPO and adjacent marginal seas still raises social concerns about potential health risks, e.g. associated with seafood consumption and fishing industry.

Recent studies have observed that ^137^Cs can disperse through the Subtropical Mode Water (STMW) and Central Mode Water (CMW) in the western NPO^9,15^. STMW is formed right south of the Kuroshio Current and Extension (between 132° E and near the dateline) by deep vertical convection in winter^16–18^. CMW forms north of the Kuroshio Extension (around 36–41° N, 160° E–165° W), hence is characterised by colder temperatures and a deeper layer (250–500 m)^19^. These two water masses (i.e., STMW and CMW) circulate clockwise beneath the surface of the western NPO^20^. When plotted against depth, the vertical profiles of ^137^Cs activity concentrations recorded at 20° N, 165° E in 2002 exhibited two distinct peaks: the first one at a potential density (σ_θ_) of 25.5 kg m^−3^ (corresponding to the σ_θ_ range of STMW) and the second one at a σ_θ_ of 26.0 kg m^−3^ (corresponding to the σ_θ_ range of CMW)^3^. The subsequent injection of FDNPP-derived radionuclides resulted in a contamination plume spreading eastward and merging with the Oyashio Current; hence the incorporation of radiocesium in the formation of STMW and CMW and its current value as a tracer of ocean circulation^4^.

Both water masses (i.e., STMW and CMW) extend clockwise to the western boundary of the NPO, where the Kuroshio is most intense. Branches of the Kuroshio Current intrude into the northern South China Sea (SCS), southern Taiwan Strait, and even the southern East China Sea (ECS). Inomata et al.^6^ estimated that 5.0% of the total amount of FDNPP-derived ^137^Cs in the STMW entered into the Sea of Japan before 2016 through clockwise spreading in the western NPO, ECS, and the Sea of Japan. Several studies have reported the intrusion of radiocesium into the western NPO but also into the adjacent marginal seas after the FDNPP event^4,5,21^. However, seawater ^137^Cs activity concentrations have hardly been investigated in the Kuroshio east of Taiwan and the Taiwan Strait, particularly after the 2011 event.

Subtropical marginal seas of the western NPO are characterized by a monsoon cycle and seasonal coastal currents. For instance, the Taiwan Strait is affected by the northeasterly monsoon in the winter and the southwesterly monsoon in the summer^22,23^. As a result, the northern half of the Taiwan Strait is characterized by a southward-flowing coastal current in the winter while the southern half of the Taiwan Strait is characterized by a warm, northward-flowing current reinforced by the Kuroshio intrusion branch in the summer^24–27^. Furthermore, there are well documented upwelling regions in the study area, such as the waters off northeastern Taiwan^28,29^, several regions in the Taiwan Strait^30,31^, and regions in the northern SCS^32–34^. However, to the best of our knowledge, the effects of seasonality and upwelling on the fate of radiocesium in seawater in this study area remain unclear.

This study reports the activity concentration of ^134^Cs and ^137^Cs over the shelf break of the western NPO and examines the fates of these radionuclides on the shallow continental shelf. The ^134^Cs and ^137^Cs activity concentrations were measured on samples collected in near-surface, subsurface or deeper waters in the Kuroshio region east of Taiwan and the Taiwan Strait between 2018 and 2019. The origin of the ^137^Cs maximum in the subsurface/deep waters and the factors controlling seasonal variations in ^137^Cs activity concentration in the near-surface waters are discussed.

## Methods

**Figure 1:**
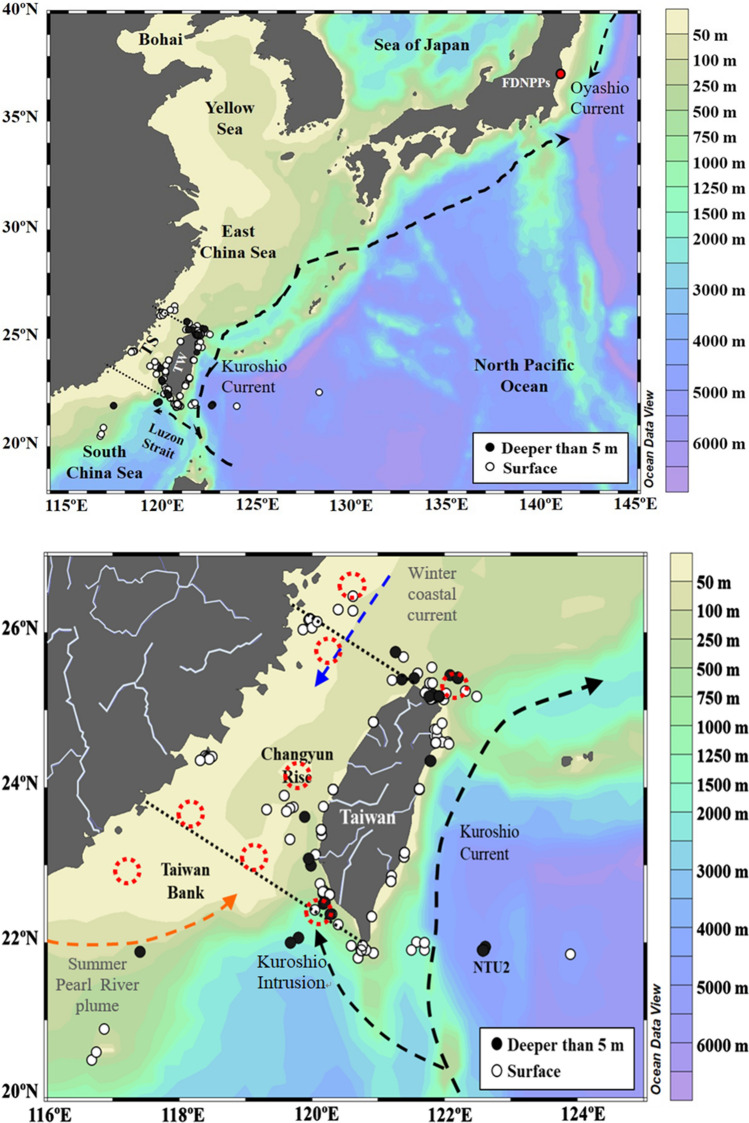
Sampling area. The Taiwan Strait and the Kuroshio east of Taiwan are critical regions in the western North Pacific Ocean (NPO). (**a**) Surface (solid markers) and subsurface and deep-water (open markers) samples were collected in the study area from 2018 to 2019. (**b**) The two dashed lines indicate the northern and southern boundaries of the Taiwan Strait used in this study. Red dashed circles are potential upwelling sites positioned using the temperature and chlorophyll-a data by Liu et al.^31^. This image was created by Ocean Data View (Version 4.7.5) (Schlitzer, R., Ocean Data View, https://odv.awi.de, 2016).

From 2018 to 2019, surface (< 5 m), subsurface (5–200 m), and deep seawater (200–1000 m) samples were collected at sites in the Kuroshio east of Taiwan and the Taiwan Strait (Fig. 1). Surface seawater samples (40 or 60 L) were collected mostly from fishing boats by using cleaned 20-L tanks. Subsurface samples were taken by using Niskin bottles mounted on a Conductivity–Temperature–Depth (CTD) rosette, which recorded temperature, salinity (from conductivity), and water depth (from pressure) onboard R/Vs Ocean Researcher I, II, and III. Sampling locations are shown in Fig. 1. Each 20-L sample was acidified using hydrochloric acid (11 M HCl, 100 mL) and was kept at room temperature (~ 15–30 °C) until it was transported to the Radian Monitor Center, Atomic Energy Council, Kaohsiung, Taiwan. Radiocesium was pre-concentrated by adsorption onto ammonium molybdophosphate (AMP)^35,36^ and counted using a high-purity germanium (HPGe) detector with lead shielding. Each 40-L or 60-L sample was counted for 200,000 s or 120,000 s, respectively. The detection limits of both ^134^Cs and ^137^Cs were 0.5 Bq m^−3^. In this study, we corrected all ^137^Cs activity concentrations to January 1st, 2020. This date was also applied to data in earlier studies for comparison purposes.

Total alkalinity (TA) samples were collected together with nearly 61% of radiocesium samples (92% of 49 subsurface and deep waters, and 55% of 242 near-surface waters). TA water samples were taken into 250-mL borosilicate glass bottles and poisoned immediately with 100-µL saturated HgCl_2_ solution to eliminate biological activities. We followed the open-cell Gran titration method with a temperature-controlled, semi-automated titrator (AS-ALK2 Apollo Scitech) to determine TA value of each sample. TA measurements were referenced against certified reference materials from A. G. Dickson’s laboratory at Scripps Institution of Oceanography with a precision of 0.1%^37^. TA is usually treated as a conservative tracer. We used it to determine the water sources and possible mixing processes between fresh water and seawater by normalizing TA values to salinity 35 (NTA = (TA/S) × 35)^38^.

As variables can be collectively controlled by major oceanographic mechanisms, principal component analysis (PCA)^39,40^ serves as a multivariate analysis tool to catch the major features of a dataset. PCA can analyze inter-correlations among these variables and reduces the dimension of a dataset. The major factors affecting this dataset is thus obtained. The result is displayed as a subset of new, independent (orthogonal) variables which are referred to as dimensions. The higher the coordinates of a dimension are, the greater the amount of co-variability among the original variables this dimension explains. The results were presented graphically as plots with each dimension and length represented the relationship and weight to the principal components, correspondingly. We applied PCA using R software^41^ on our data set for which four variables had been determined: salinity, temperature, σ_θ_, and ^137^Cs activity concentrations from the surface layer to a depth of 400 m. The oceanographic context is then used to interpret the meaning of each dimension.

## Results

### Surface water properties and the distribution of ^134^Cs and ^137^Cs

**Figure 2:**
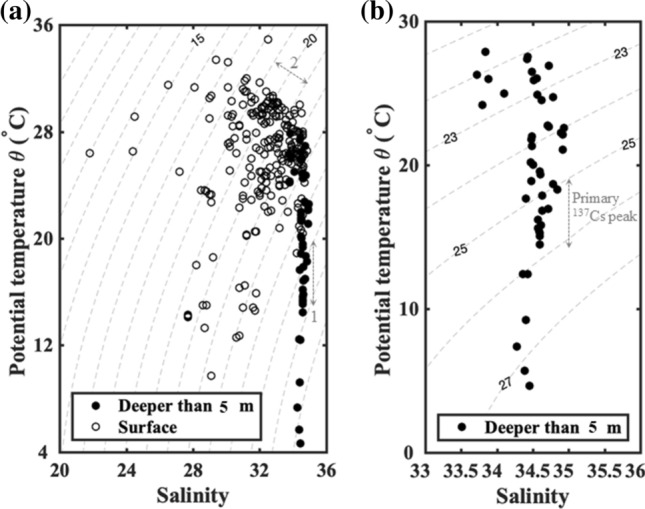
Temperature and salinity diagram. (**a**) Surface waters showed large variations in temperature and salinity. (**b**) Deep waters demonstrated salinities higher than 33.5 and represent the σ_θ_ range of the Subtropical Mode Water (STMW) and Central Mode Water (CMW) (25.1–26.2 kg m^−3^). Labels “1” and “2” in (**a**) represent the σ_θ_ range of primary and secondary ^137^Cs peaks, respectively.

**Figure 3:**
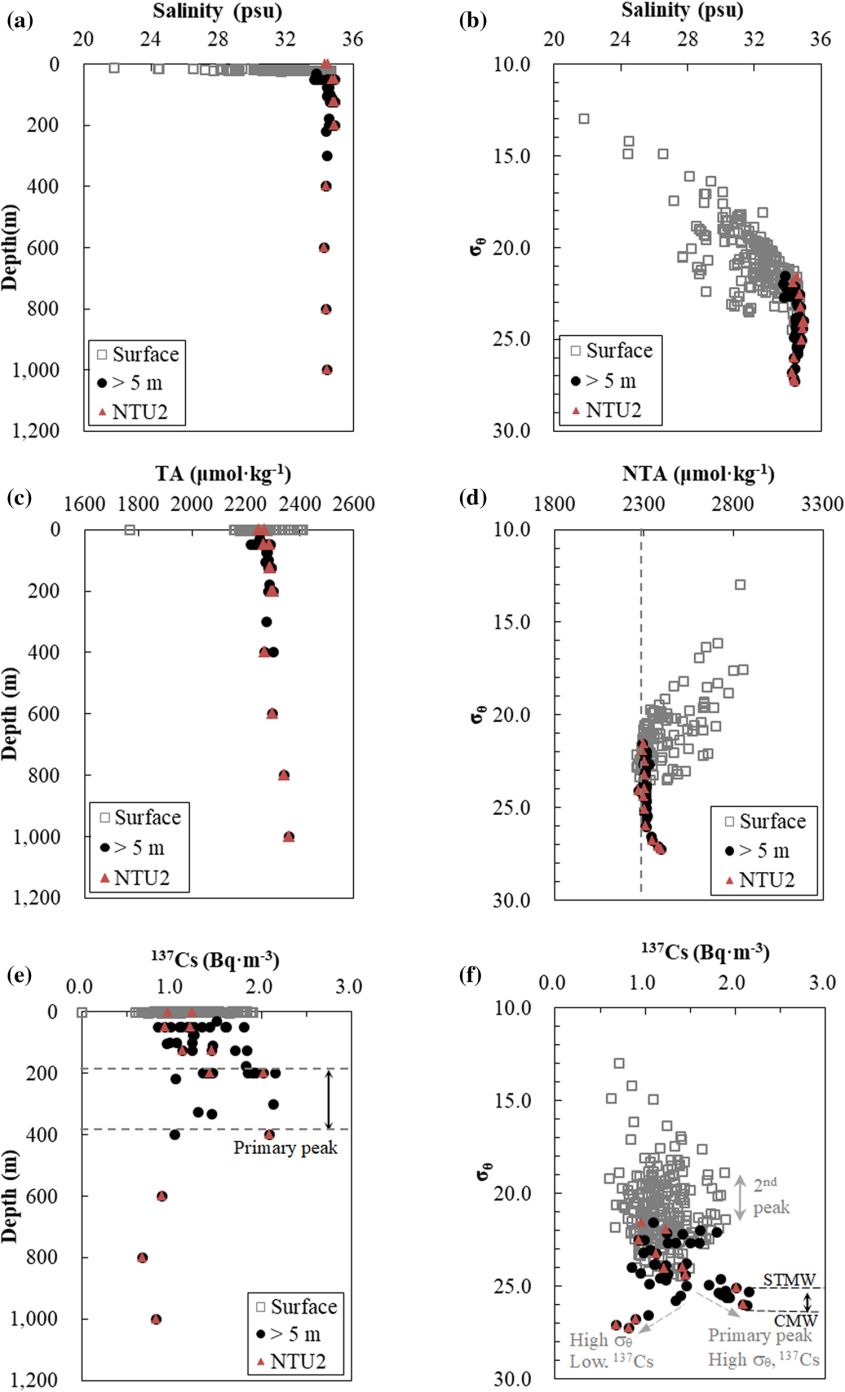
Vertical distributions of salinity, TA, NTA, and ^137^Cs concentration. In the subsurface and deep waters, where (**a**) salinities varied from 33.7 to 34.9 long the vertical profile, (**b**) TA values varied from 2284 to 2360 μmol kg^−1^, (**c**) ^137^Cs activity concentrations displayed the primary peak at a depth range of 200–400 m, and where (**d**) σ_θ_ varied from 25.2 to 26.1 kg m^−3^, (**e**) NTA was 2309 ± 5 μmol kg^−1^, (**f**) the primary peak of ^137^Cs was observed. (**d**) NTA with low σ_θ_ values deviated from 2309 in the near-surface waters. (**f**) The secondary peak of ^137^Cs activity concentration displayed σ_θ_ ranging from 19 to 22 kg m^−3^ in the near-surface waters. All panels share the same legend. Data from NTU2 were noted with triangle markers in corresponding solid circles.

Surface water samples were obtained from depths of less than 5 m, with temperatures of 9.7–34.9 °C and salinities of 21.8–34.2 psu (Figs. 2 and 3a). Salinity, TA, and σ_θ_ displayed large variations in surface waters (Fig. 3). Salinities were comprised between 35 and 21 psu and σ_θ_ was usually lower than 24 kg m^−3^ (Fig. 3a,b), indicating mixing between seawater and fresh water. The average TA in surface waters was 2243 ± 38 μmol kg^−1^. NTA values associated with low σ_θ_ values, i.e., surface waters, deviated significantly from the average value determined in subsurface and deep waters (2309 μmol kg^−1^) (Fig. 3d), implying that TA values in surface waters were likely to be affected by additional river TA sources.

In our samples, ^134^Cs activity concentrations were under the detection limit (0.5 Bq m^−3^), and ^137^Cs activity concentrations ranged from 0.5 to 2.0 Bq m^−3^ (Fig. 3e,f), with an average of 1.2 ± 0.3 Bq m^−3^ in the surface water (Supplementary Fig. S1). We also noticed a peak in the surface water ^137^Cs vertical profile (Fig. 3f), with the maximum value ranging from 1.95 to 1.96 Bq m^−3^ (σ_θ_ = 18.8 to 21.4 kg m^−3^).

**Figure 4:**
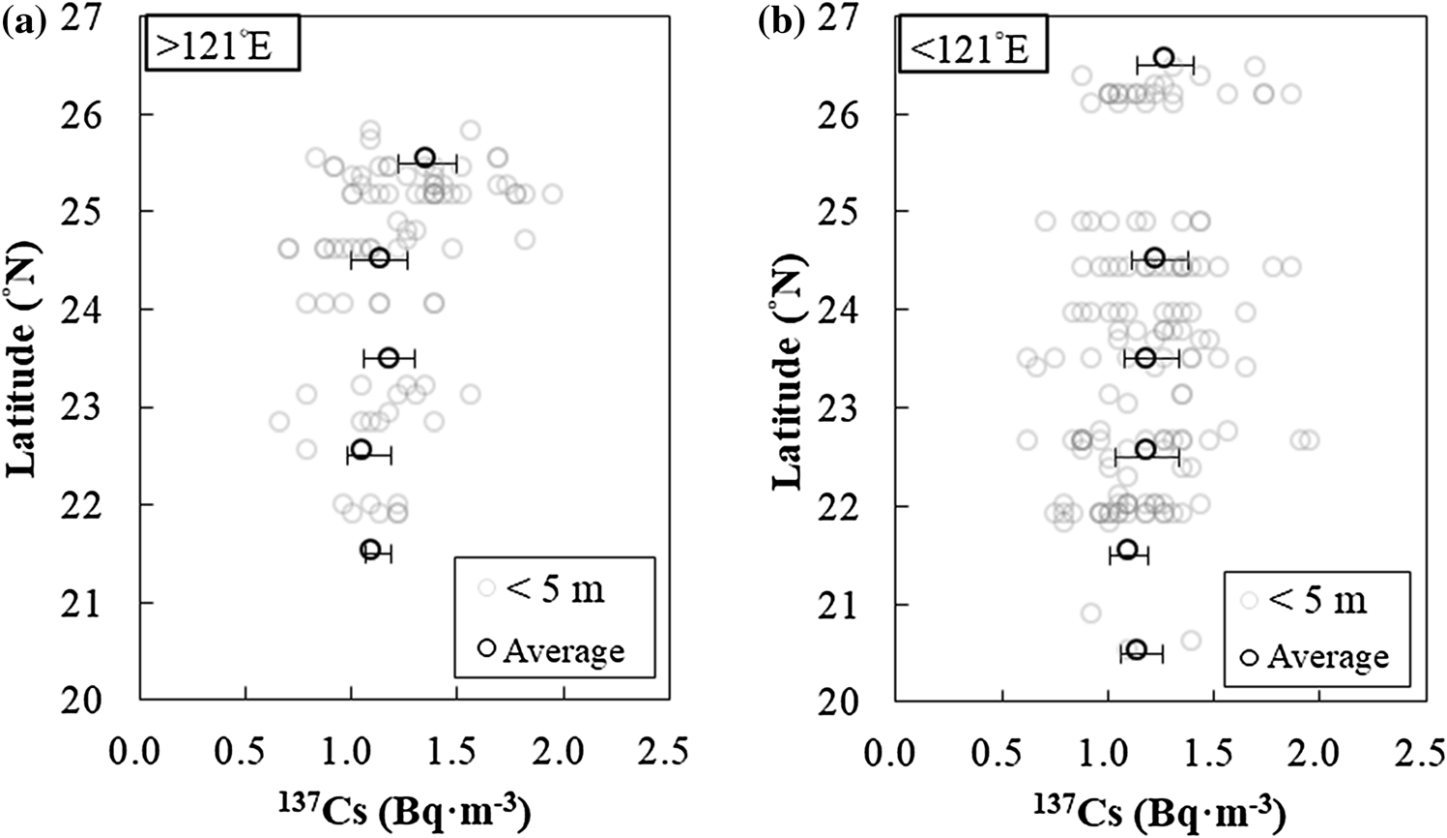
Latitude to ^137^Cs relationships in the near-surface waters. To the east of 121° E (**a**), the binned ^137^Cs activity concentration in one-degree latitude bands from 25 to 26° N was higher than the one from 21 to 22° N (black squares). (**b**) The binned ^137^Cs values in each latitude were close to each other to the west of 121° E.

We arbitrarily divided the study area into geographic sectors and listed the average ^137^Cs values for each sector in Table 1. The average values of ^137^Cs activity concentrations in each sector were similar, with ^137^Cs = 1.2 ± 0.3 Bq m^−3^ in the Taiwan Strait and 1.2 ± 0.2 Bq m^−3^ in the Kuroshio and its adjacent waters (Table 1). In the surface waters, the ^137^Cs values binned in one-degree latitude bands were higher between 25 to 26° N than the ones between 21 to 22° N in the Kuroshio and its adjacent waters (approximately to the east of 121° E in our study area) (Fig. 4a). Binned ^137^Cs activity concentrations were close to each other to the west of 121° E (Fig. 4b).

**Table 1:** Average ^137^Cs values for the Taiwan Strait and the Kuroshio Current (KC) east of Taiwan (2018–2019) in Bq m^−3^.

Sub-division	^137^Cs	
	Mean	STD^a^
Taiwan strait	1.2	0.3
KC and adjacent water	1.2	0.2

^a^STD represents the standard deviation of the corresponding mean values.

**Figure 5:**
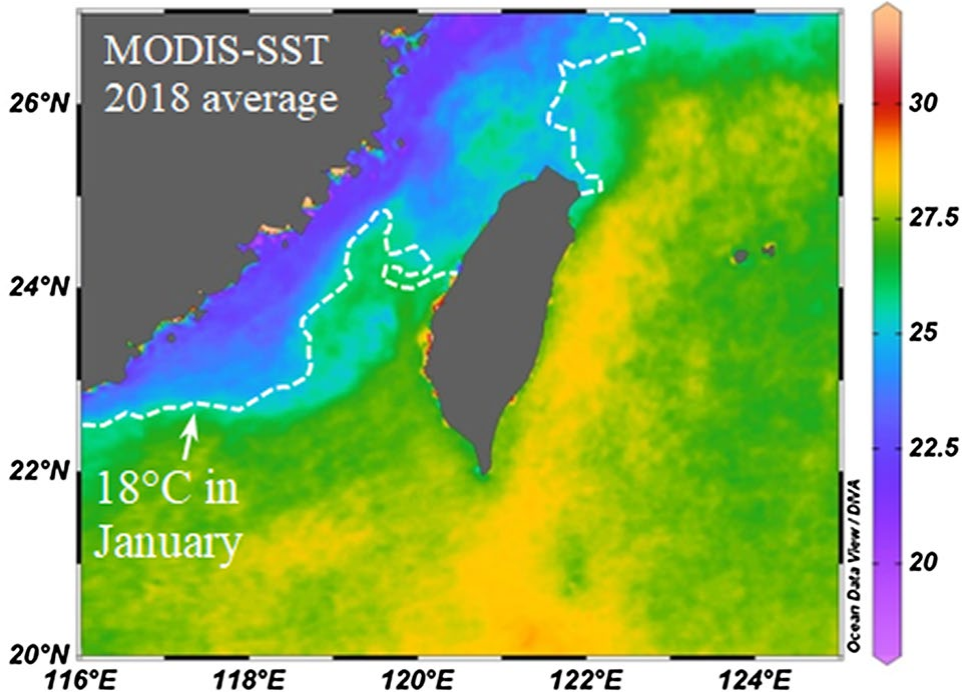
Sea surface distributions of seawater temperature. SST is an important factor while understanding the oceanic condition. High SST (MODIS 2018) was detected in the Kuroshio east of Taiwan and its intrusion into the southeastern Taiwan Strait. Low SST was identified along the coastline from the East China Sea (ECS) to the western side of the Taiwan Strait. The white dashed line indicates the contour line of 18 °C obtained from January. Monthly SST data was collected from AQUA-MODIS with a spatial resolution of 1° × 1° (https://neo.sci.gsfc.nasa.gov/view.php?datasetId=MYD28M). This data was accessed on July 3, 2021. This image was created by Ocean Data View (Version 4.7.5) (Schlitzer, R., Ocean Data View, https://odv.awi.de, 2016).

The sea surface temperature (SST) distribution in 2018 displayed a general pattern, where SST values of less than 25 °C were found in the southern ECS and the northern Taiwan Strait while SST values over 27 °C were common for the Kuroshio and the Luzon Strait (Fig. 5). The SST in the northern Taiwan Strait and southern ECS showed strong seasonal variations, displaying SST over 25 °C during the summer-like months (i.e., July–September) and less than 25 °C during winter-like months (i.e., January–March) (Supplementary Fig. S2). The SST in shelf waters of the northern Taiwan Strait displayed stronger seasonal variations than those in the pelagic Kuroshio waters east of Taiwan.

**Figure 6:**
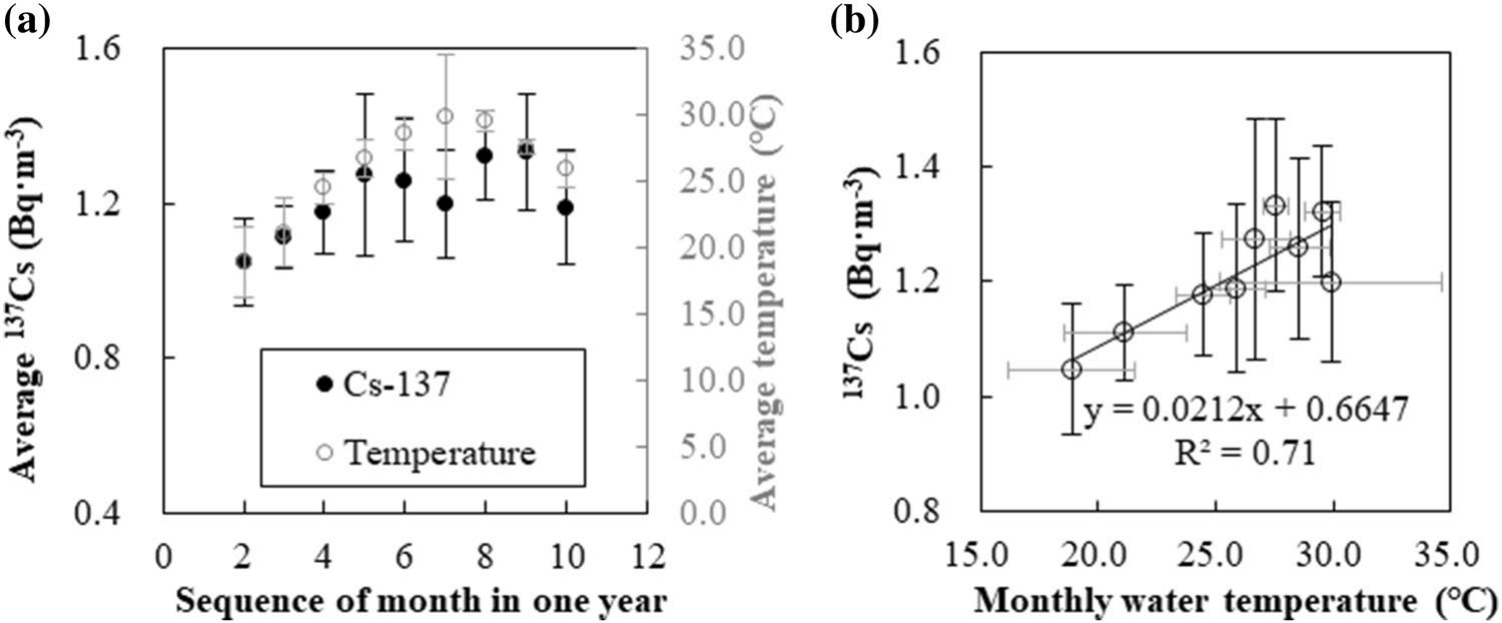
Monthly variations in sea-surface ^137^Cs activity concentration in the Taiwan Strait and southern ECS. (**a**) ^137^Cs activity concentration and corresponding seawater temperature were measured at the study sites on both sides of the Taiwan Strait and the waters off northern Taiwan (refer to Fig. 1b). Both parameters displayed seasonal variations: low in winter and high in summer. (**b**) The monthly ^137^Cs activity concentration was statistically correlated to its corresponding seawater temperature.

In the shelf waters, including the Taiwan Strait and the waters off northern Taiwan, the average ^137^Cs activity concentration was statistically higher in August than in February (two-tailed *t*-test, p < 0.05) (Fig. 6a). A statistically significant relationship was observed between monthly variations of ^137^Cs and temperature (Fig. 6b). Monthly values of ^137^Cs (Fig. 6a) increased from winter to summer and again started to decrease in fall, implying a seasonal cycle of ^137^Cs in surface shelf waters.

### Subsurface and deep water properties and distributions of ^134^Cs and ^137^Cs

Subsurface/deep water samples were taken from depths of 5–1000 m, with temperatures of 4.7–28.2 °C, and salinities over 21.0 to 34.0 psu (Fig. 2). The T–S characteristics of the subsurface and deep waters (Fig. 2b) of the Kuroshio-influenced region of the NPO covered the signals of STMW (σ_θ_: ~ 25.6 kg m^−3^), Kuroshio Tropical Water (KTW, T = 17.0 °C, S = 34.6 psu), and CMW (σ_θ_ ~ 26.1 kg m^−3^). TA values in the subsurface and deep waters varied in a narrow range (Fig. 3c). The average and standard deviation of NTA value for waters between 5 to 400 m was 2309 ± 5 μmol kg^−1^ (Fig. 3d).

At station NTU2 in the Kuroshio region, ^134^Cs activities were below the detection limit (0.5 Bq m^−3^), and ^137^Cs activities were lower than 2.5 Bq m^−3^ from the surface to a depth of 1000 m (Fig. 3e). Moreover, two layers of elevated ^137^Cs activities were observed: ^137^Cs activities were mostly higher than 1 Bq m^−3^ from 0 to 400 m and also higher than 2 Bq m^−3^ from 200 to 400 m. By contrast, they were lower than 1 Bq m^−3^ from 600 to 1000 m (Fig. 3f). A synthesis of these results showed that low ^137^Cs activity concentrations (1.5 to 0.6 Bq m^−3^) corresponded to the high σ_θ_ (> 25 kg m^−3^) waters between 400 and 1000 m (Fig. 3e,f).

## Discussion

**Figure 7:**
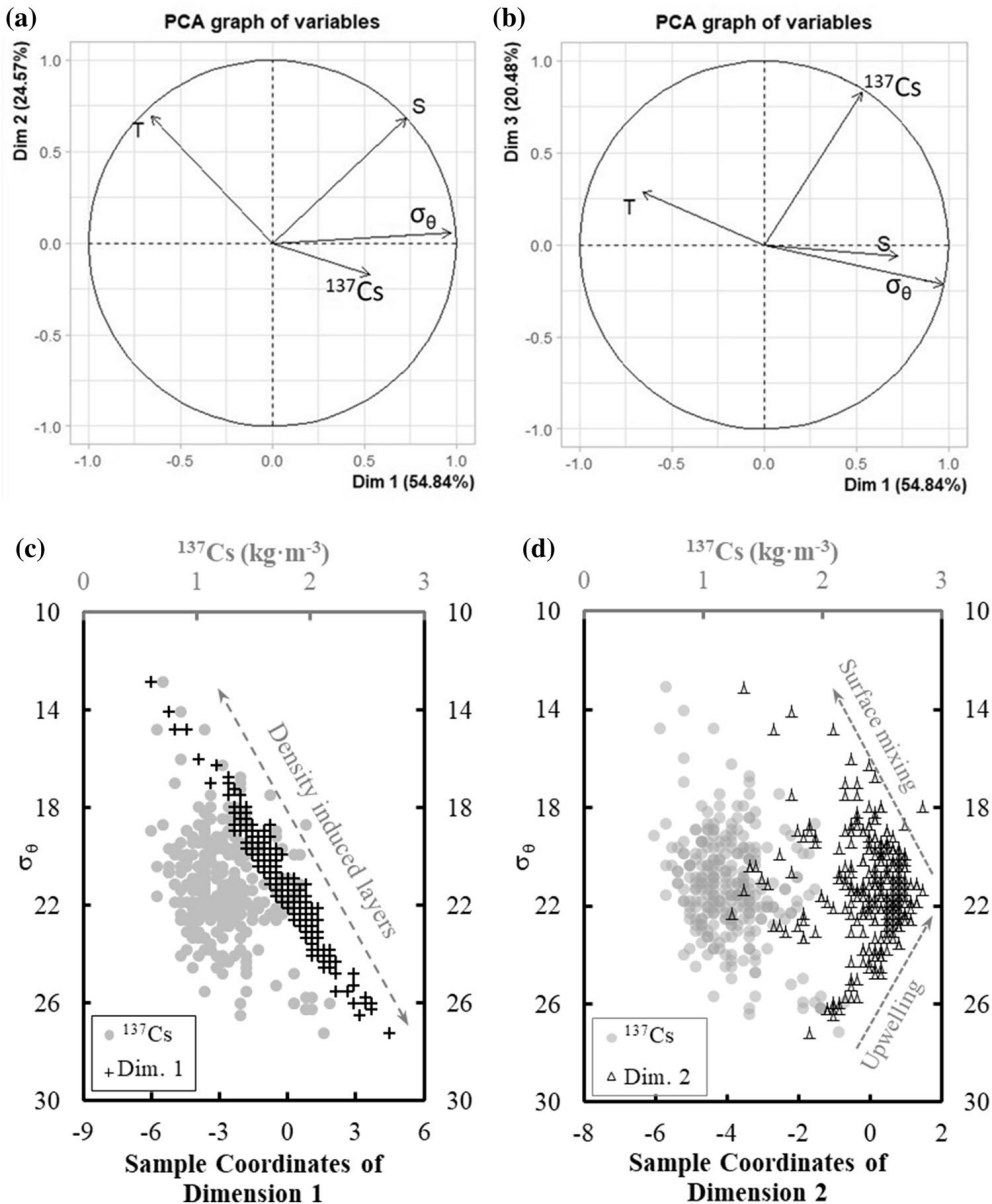
Results of principal component analysis (PCA) and sample coordinates. (**a**,**b**) σ_θ_ dominated variations in Dimension 1; increasing temperature and salinity dominated variations in Dimension 2; and ^137^Cs dominated variations in Dimension 3. (**c**) The coordinates of individual results in Dimension 1 were consistent with the primary peak of ^137^Cs defined along the stratified water column. (**d**) The peak coordinate of Dimension 2 was consistent with the secondary ^137^Cs peak along the σ_θ_ gradient.

The first three dimensions of the PCA results explained 91% of all variations, including sample temperature, salinity, and σ_θ_, and ^137^Cs activity concentration above the depth of 400 m. Dimension 1 explained 54% of the variations (Fig. 7a,b) and was dominated by σ_θ_, temperature, and salinity (Table 2). σ_θ_, salinity, and ^137^Cs were positively correlated with Dimension 1 while the temperature was negatively correlated (Fig. 7a). We suggest that Dimension 1 represents density-induced water layer distributions and explains the primary peak of ^137^Cs in the deep waters. This suggestion is consistent with the fact that σ_θ_ is positively correlated with the variables that have a high loading on Dimension 1 (Fig. 7c) and is a marker for the two layers of elevated ^137^Cs in the top 400 m of the water column.

**Table 2:** Principal components.

	Dim. 1	Dim. 2	Dim. 3
Temperature	− 0.659	0.696	0.284
Salinity	0.728	0.682	− 0.061
σ_θ_	0.974	0.055	− 0.213
^137^Cs activity	0.530	− 0.174	0.830

The highest coordinate of Dimension 2 corresponds to the secondary peak of ^137^Cs in the surface waters (Fig. 7d), implying that Dimension 2 represents a factor that led to the secondary ^137^Cs peak. The plot of Dimension 2 coordinates against σ_θ_ (Fig. 7d) can be divided into a high σ_θ_ (> 22 kg m^−3^) arm and a low σ_θ_ (< 22 kg m^−3^) arm. As both temperature and salinity were positively correlated with Dimension 2 (Fig. 7a, Table 2), we hypothesize that the high σ_θ_ arm was caused by upwelling which transports high salinity water toward the surface and reduces the thickness of the lens of warm surface water in this study area. Upwelling near the coast of Taiwan and in the Taiwan Strait is known to transport subsurface waters (as deep as 80 m to 100 m) to the near-surface layer. The vertical velocity of upwelling off northeastern Taiwan (Fig. 1)^42–45^ has been estimated to be 15 m day^−1^ on the shelf and over 40 m day^−1^ at the shelf edge^29,46^. The σ_θ_ of water with salinity = 34.3 psu, temperature = 18.3 °C, and σ_θ_ = 25.0 kg m^−3^ at a depth of 200 m, can decrease to less than 22 kg m^−3^ if the water temperature increases to 28 °C at 1 m during the upwelling process (Fig. 2a). This annual upwelling to the northeastern Taiwan may lead to the higher binned ^137^Cs between 25 and 26° N than the others to the east of 121°E in the near-surface waters (Fig. 4a). Another driving force of annual upwelling is internal tide, which can induce upwelling in the waters off northeastern Taiwan^47^ and off southern Taiwan^48–50^. Moreover, this transition σ_θ_ of 22 kg m^−3^ in Fig. 7d was also consistent with the transition range of σ_θ_ where NTA positively deviated from 2309 ± 5 μmol kg^−1^ in the near-surface water (Fig. 3d). This deviation of NTA in low σ_θ_ waters indicates freshwater inputs from terrestrial runoff. To sum up, we suggest that mixing between riverine freshwater and seawater is responsible for the lower σ_θ_ arm while upwelling drives the higher σ_θ_ arm in Fig. 7d.

Dimension 3 accounted for no more of the variance than ^137^Cs itself (Table 2, Fig. 7b), suggesting that it was controlled by the chemical characteristics of ^137^Cs. MacKenzie et al.^51^ have argued that high freshwater discharge from land can remobilize ^137^Cs from surface sediments (< 10 cm). Future work should be conducted towards a dynamic representation of radionuclide transfer among freshwater, seawater, sediment, and the biological compartments^11^.

The seasonal variation of the ^137^Cs activity concentration in the Taiwan Strait (Fig. 6a) reflects the seasonal intrusions of waters from Kuroshio intrusion, ECS, and SCS. Wu et al.^19^ corrected their ^137^Cs activity concentrations to the same date as this study, leading to a mean of 0.71 ± 0.27 Bq m^−3^ in the surface ECS and an average of 0.92 ± 0.28 Bq m^−3^ in the surface SCS. These results are consistent with our observation that southward-flowing cold waters with low ^137^Cs values intruded into the northern half of the shallow Taiwan Strait during the winter while the reverse took place during the summer (Fig. 6a)^22,24,25^. In addition to the Kuroshio offshoot transporting warm waters with higher ^137^Cs values in the summer, the Pearl river plume can also intrude into the southern Taiwan Strait during the summer^52^. It follows that the slightly lower ^137^Cs activity concentration during July indicates that the effect of the intrusion of Kuroshio is more than offset by the amount of warm water with lower ^137^Cs activity originating from the SCS.

The maximum in ^137^Cs activity concentration was observed in a specific range of σ_θ_ in the subsurface and deep waters (Fig. 3e,f), implying a lateral transport along the 125–400 m depth horizon in addition to local atmospheric fallout. Local and modern atmospheric ^137^Cs fallout can only affect the average ^137^Cs values in the surface waters (Table 1). ^137^Cs activity concentrations in the subsurface water of the study area displayed characteristics (σ_θ_ = 25.2 and 26.1 kg m^−3^) which were similar to those of STMW and CMW (σ_θ_ = 25.3 to 26.3 kg m^−3^) in NPO^4^ (Fig. 3). Some surface NTA values and also the average NTA value in subsurface waters (2309 ± 5 μmol kg^−1^) of this study were consistent with NTA values previously reported in the surface western NPO (2301 ± 9 to 2299 ± 5.4 μmol kg^−1^)^53,54^. The maximum ^137^Cs activity concentration at a depth of 300 to 400 m at 165° E before the FDNPP event should be 1.65 Bq m^−3^ (corrected to January 1st, 2020)^3^. After the FDNPP event in June/July 2012, ^137^Cs activity concentrations at depths of between 0 and 600 m in the western NPO (25 to 45° N, 165° E) were between 2.1 and higher than 8.4 Bq m^−3^ (corrected to January 1st, 2020)^4^. As the half-life of ^134^Cs is shorter than that of ^137^Cs, ^134^Cs/^137^Cs is assumed to be 1.000 at 165° E; it became 0.095 after 7.5 year (July 2012 to December 2020). Since ^137^Cs activity concentration is already low (< 2.1 Bq m^−3^) in this study area, ^134^Cs is expected to be < 0.2 Bq m^−3^ which is lower than the detection limit (0.5 Bq m^−3^) in this study.

The increase between the estimated ^137^Cs activity concentration before FDNPP (1.65 Bq m^−3^) and the maximum measured ^137^Cs value after FDNPP (2.1 Bq m^−3^) in this study (both corrected to January 1st, 2020) implies an additional ^137^Cs activity concentration of 2.1–1.65 = 0.45 Bq m^−3^ during this time period. This increase is the result of complex physical transportation from NPO to the study area. Kamidaira et al.^55^ reported that approximately 43% of FDNPP-derived ^137^Cs could be delivered to below the mixed layer through eddy processes. In addition, ^137^Cs in STMW and CMW appears to be transported clockwise toward the western boundary of the NPO^3,56–59^. This subsurface or deep-water layer corresponding to σ_θ_ = 26.7 kg m^−3^ can further rise westward and reach the shelf break surrounding Taiwan^60–66^. East of Taiwan, the Kuroshio is a swift and powerful current that can reach as deep as 400–600 m^67^. While the primary ^137^Cs maximum centered around σ_θ_ of 25.3 to 26.1 kg m^−3^ is suspected to be from lateral transportation from the NPO, there is still a research gap between pelagic studies in the NPO and shelf break data in this study area. For example, impacts of interleaving^68^ and meso-scale eddies^69^ on the cross-Kuroshio transport mechanism of ^137^Cs are still unclear. Direct evidence to constrain the origin and evolution of the ^137^Cs maxima along 165° E to this study area is needed in the future. We suggest integrating multiple chemical tracers to study the complex circulation across the pelagic NPO to its western shelf boundary in the future.

## Supplementary Information

Supplementary Information.
