# Supplementary material for: Radiocesium in the Taiwan Strait and the Kuroshio east of Taiwan from 2018 to 2019
Source: Sci Rep. 2021 Nov 17;11:22467. doi: 10.1038/s41598-021-01895-y (PMC8599701; doi:10.1038/s41598-021-01895-y)
Supplement: Supplementary file 1 — Supplementary Information. [file 41598_2021_1895_MOESM1_ESM.docx]

**Supplementary information for Radiocesium in the Taiwan Strait and the Kuroshio east of Taiwan from 2018 to 2019**

Wei-Jen Huang^1*^, Ming-Ta Lee^2^, Kuei-Chen Huang^1^, Kai-Jung Kao^1^, Ming-An Lee^3,4^, Yiing-Jang Yang^5^, Sen Jan^5^, Chen-Tung Arthur Chen^1*^

^1^Department of Oceanography, National Sun Yat-sen University, Kaohsiung, Taiwan

^2^The Radiation Monitoring Center, Atomic Energy Council, Kaohsiung, Taiwan

^3^Department of Environmental Biology and Fisheries Science, National Taiwan Ocean University, Keelung, Taiwan

^4^Center of Excellence for Ocean Engineering, National Taiwan Ocean University, Keelung 20224, Taiwan

^5^Institute of Oceanography, National Taiwan University, Taipei, Taiwan

**Abstract**

This supplementary information includes two figures and one table.


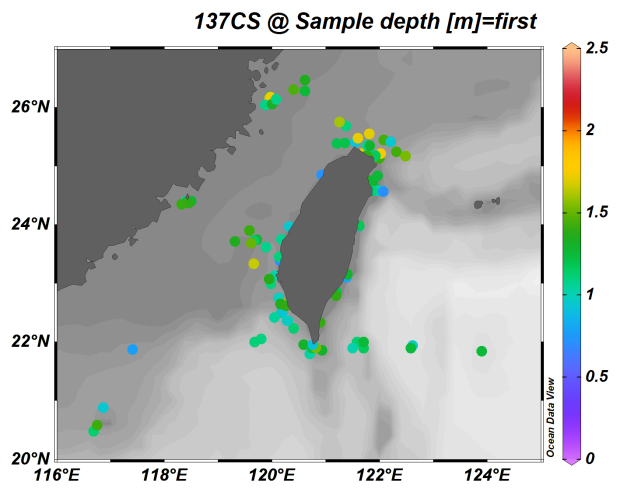


Surface ^137^Cs

(Bq∙m^-3^)

**Figure S1. Sea surface distributions of ^137^Cs.** The ^137^Cs values from the sea surface waters were scattered with a few values close to 2 Bq∙m^-3^ in the waters off northern Taiwan. This image was created by Ocean Data View (Version 4.7.5) (Schlitzer, R., Ocean Data View, https://odv.awi.de, 2016).


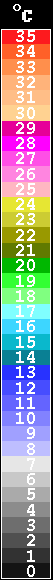

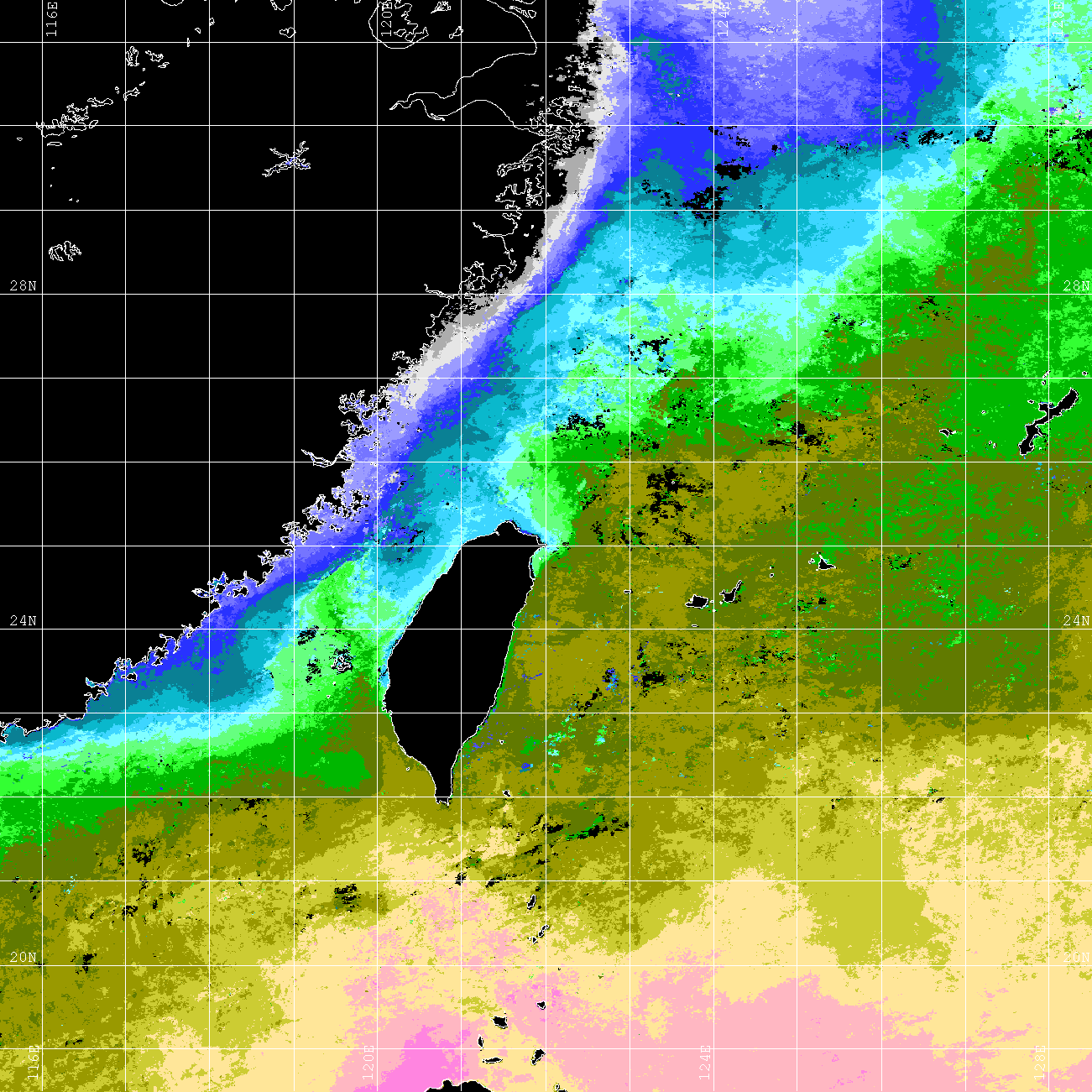

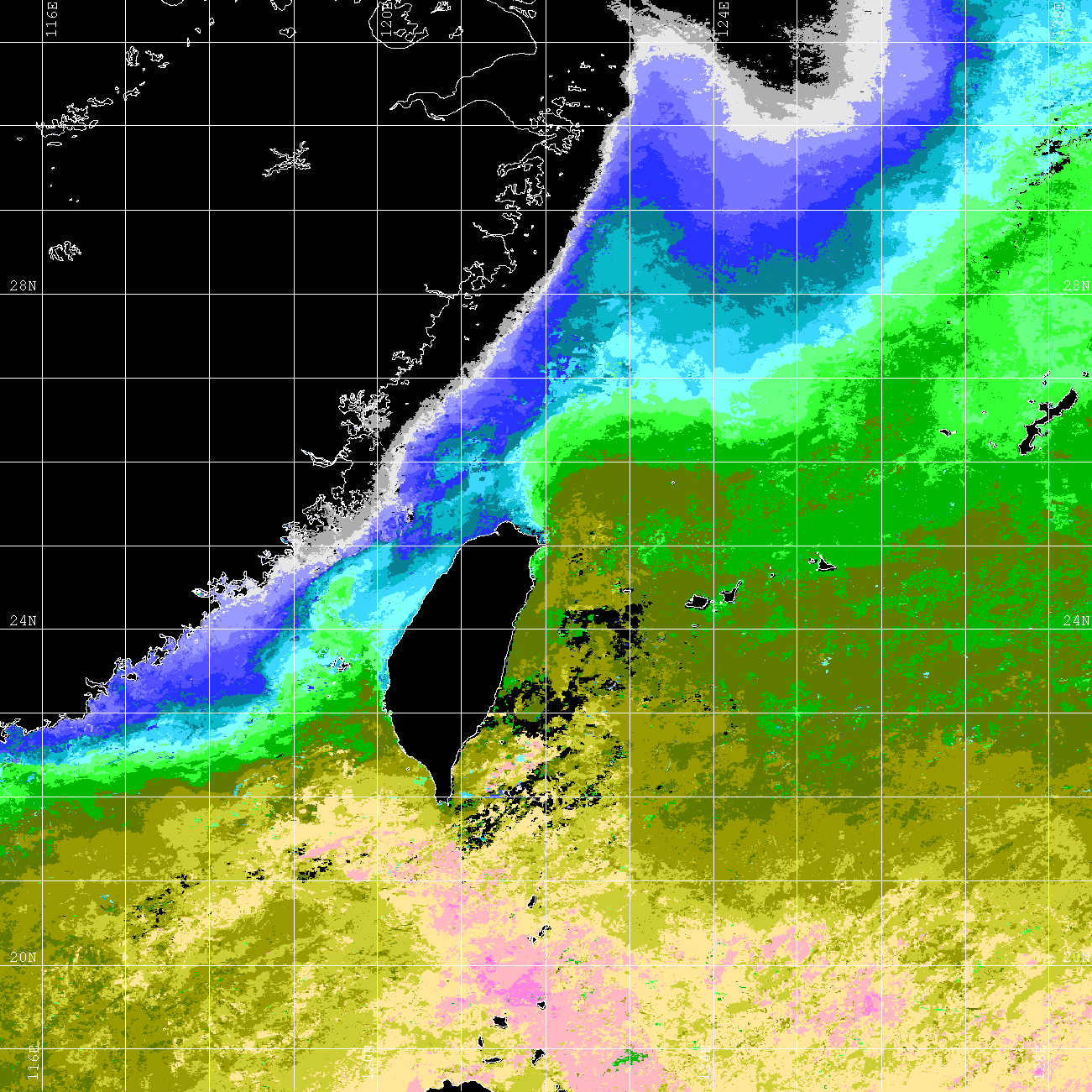

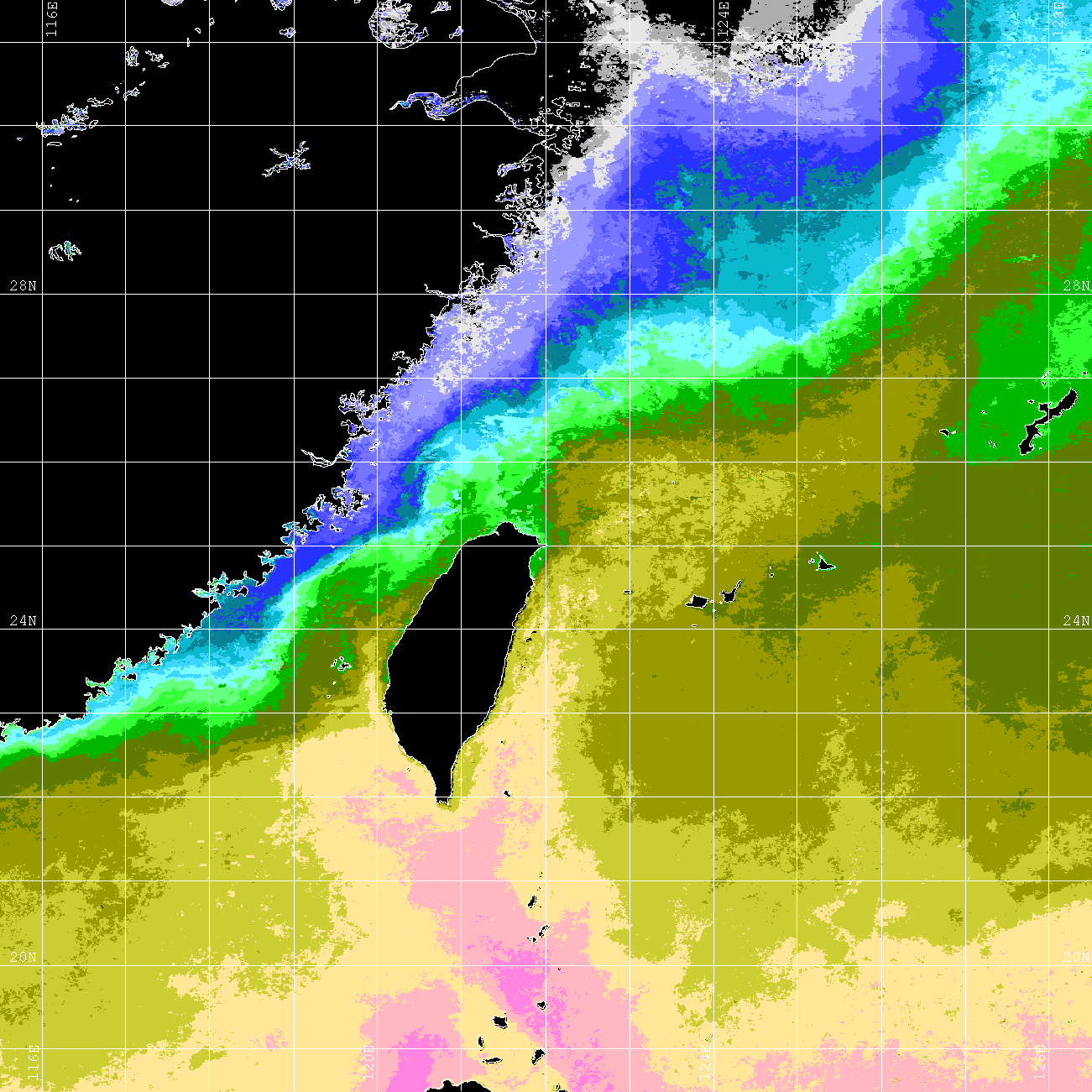

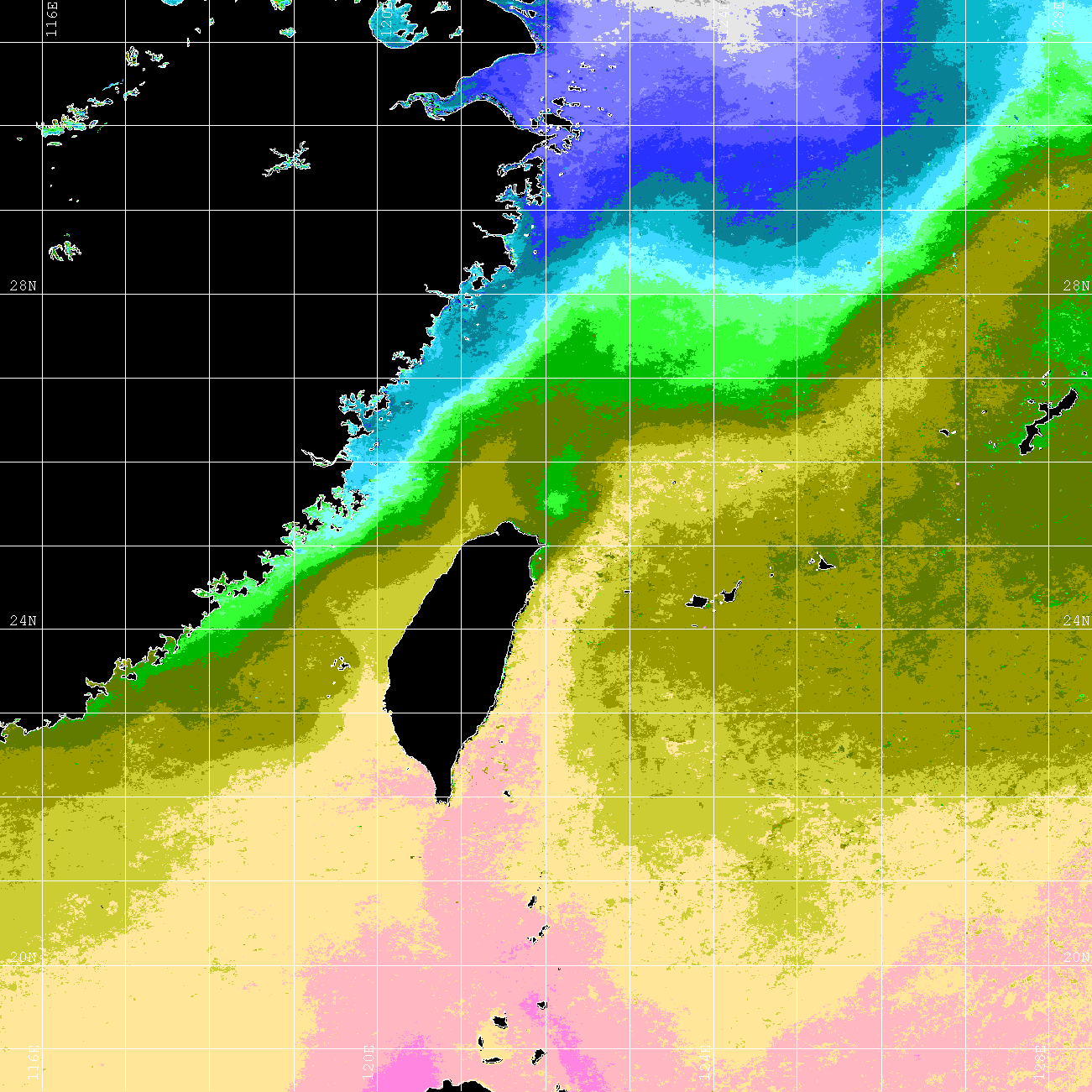


d) April

c) March

b) February

a) January


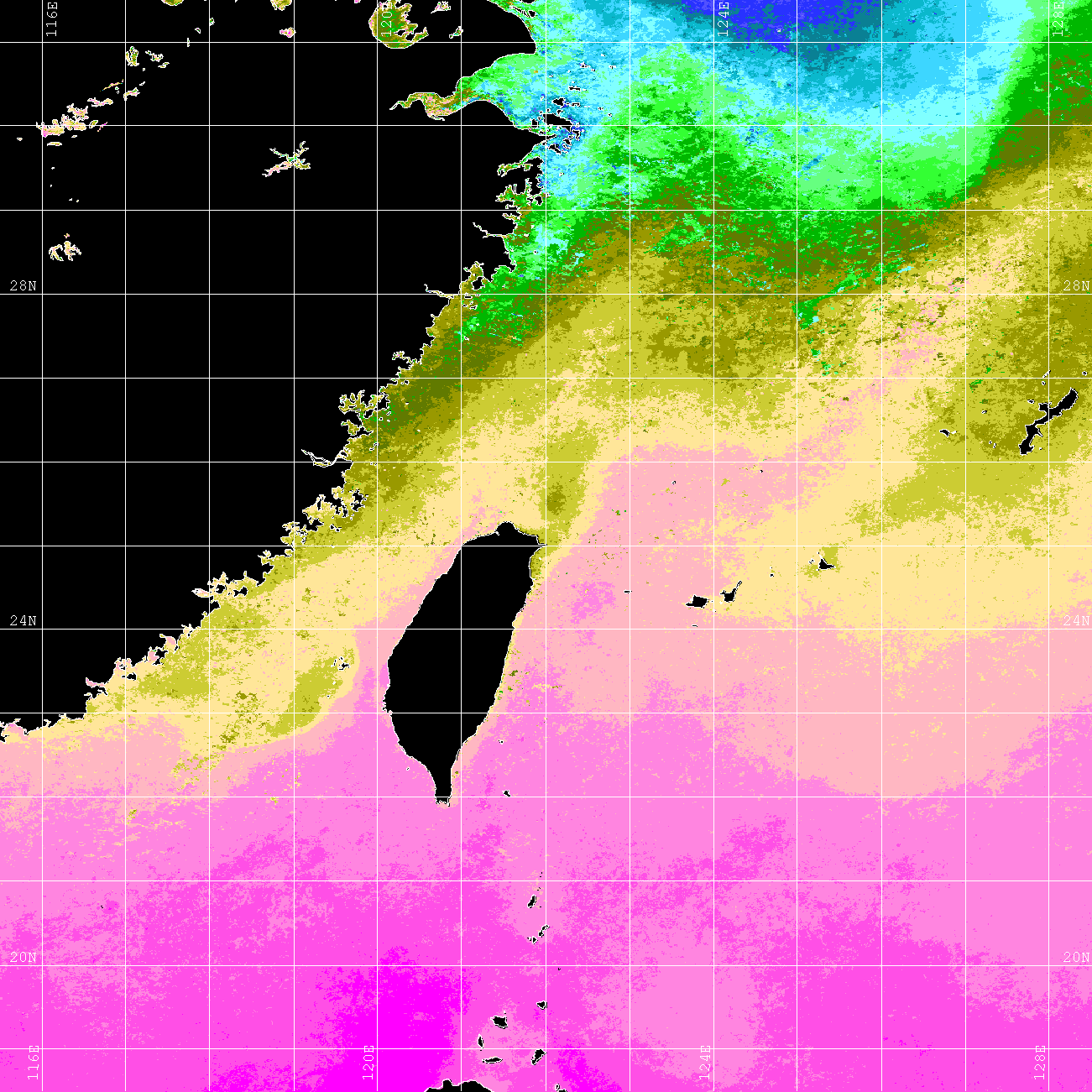

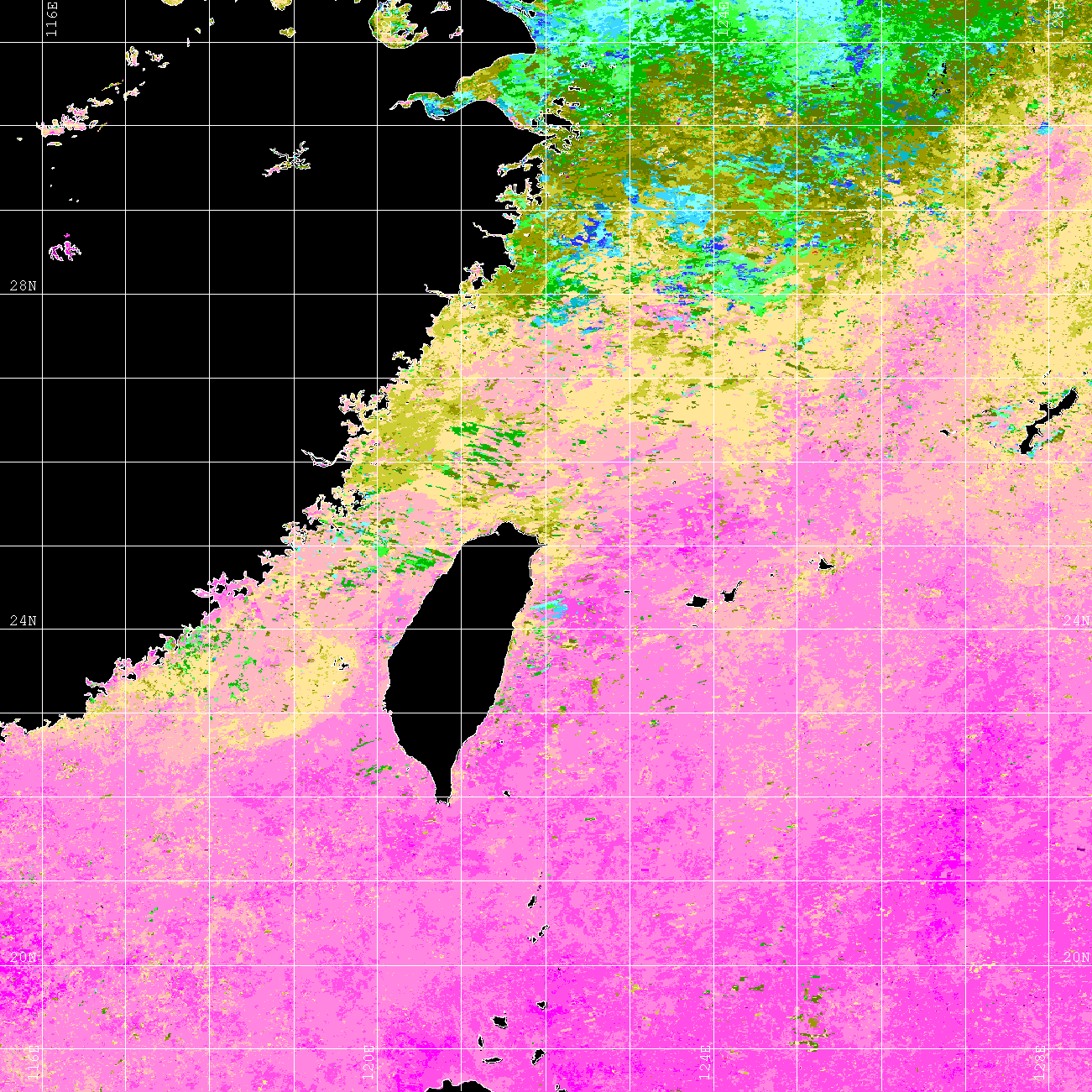

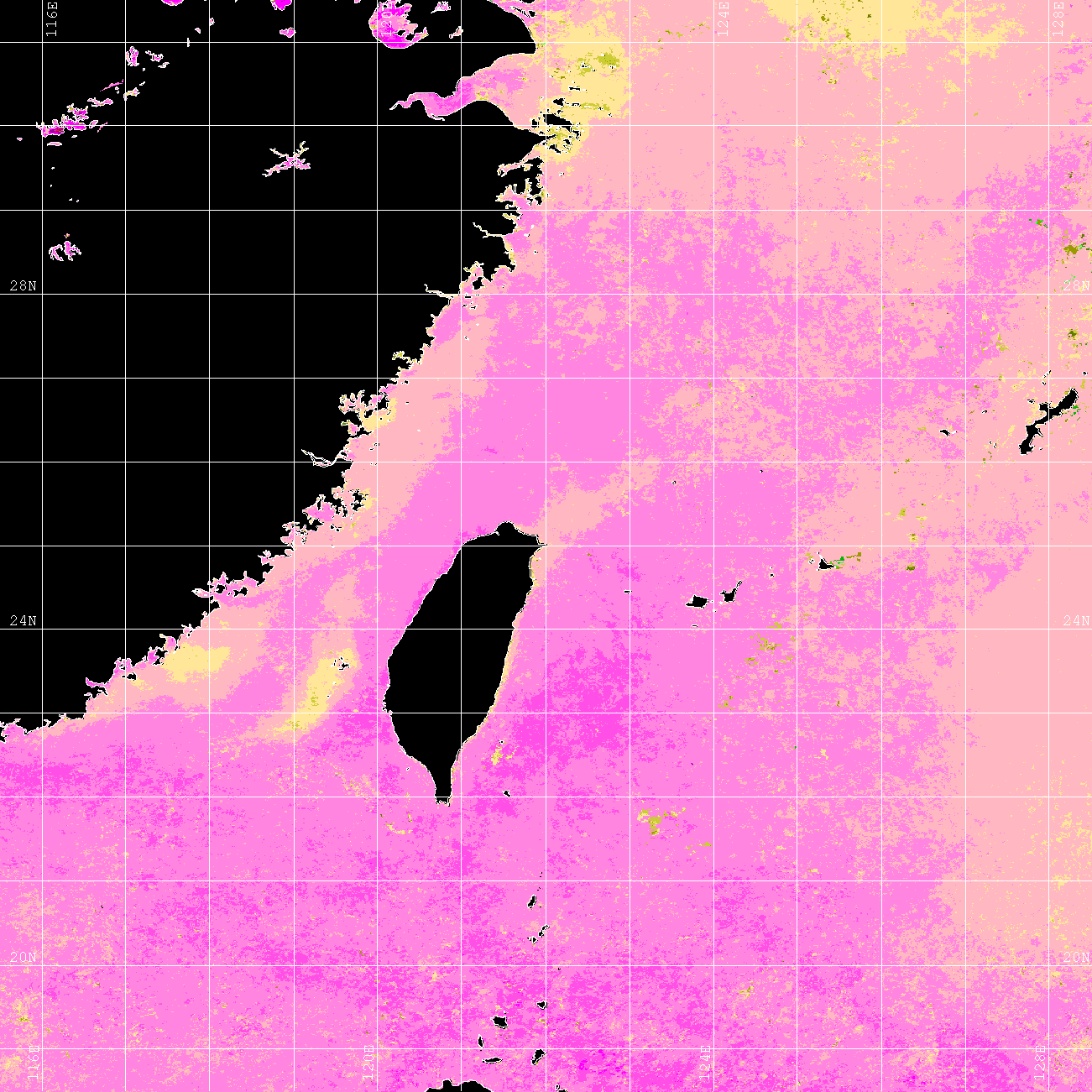

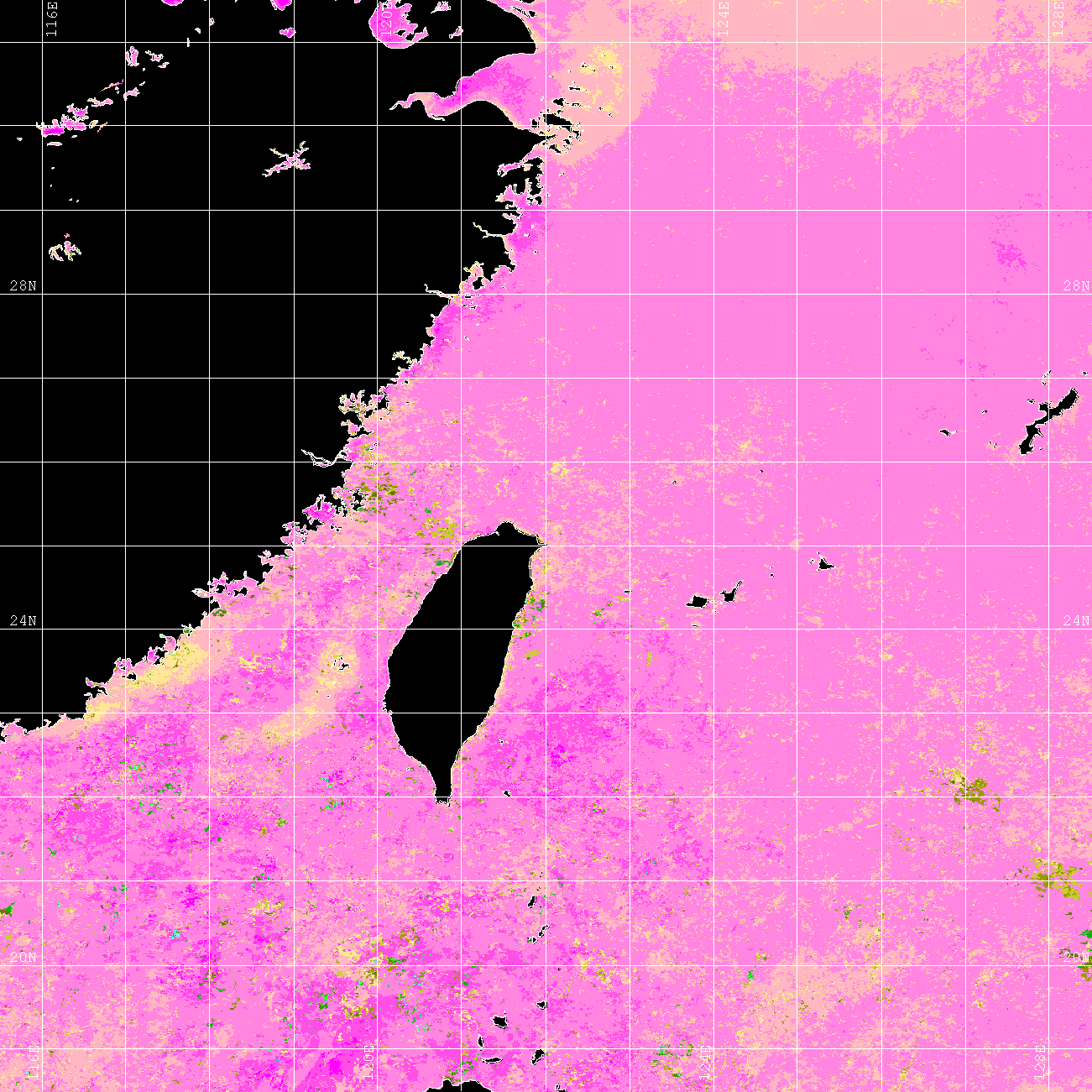


f) June

h) August

g) July

e) May


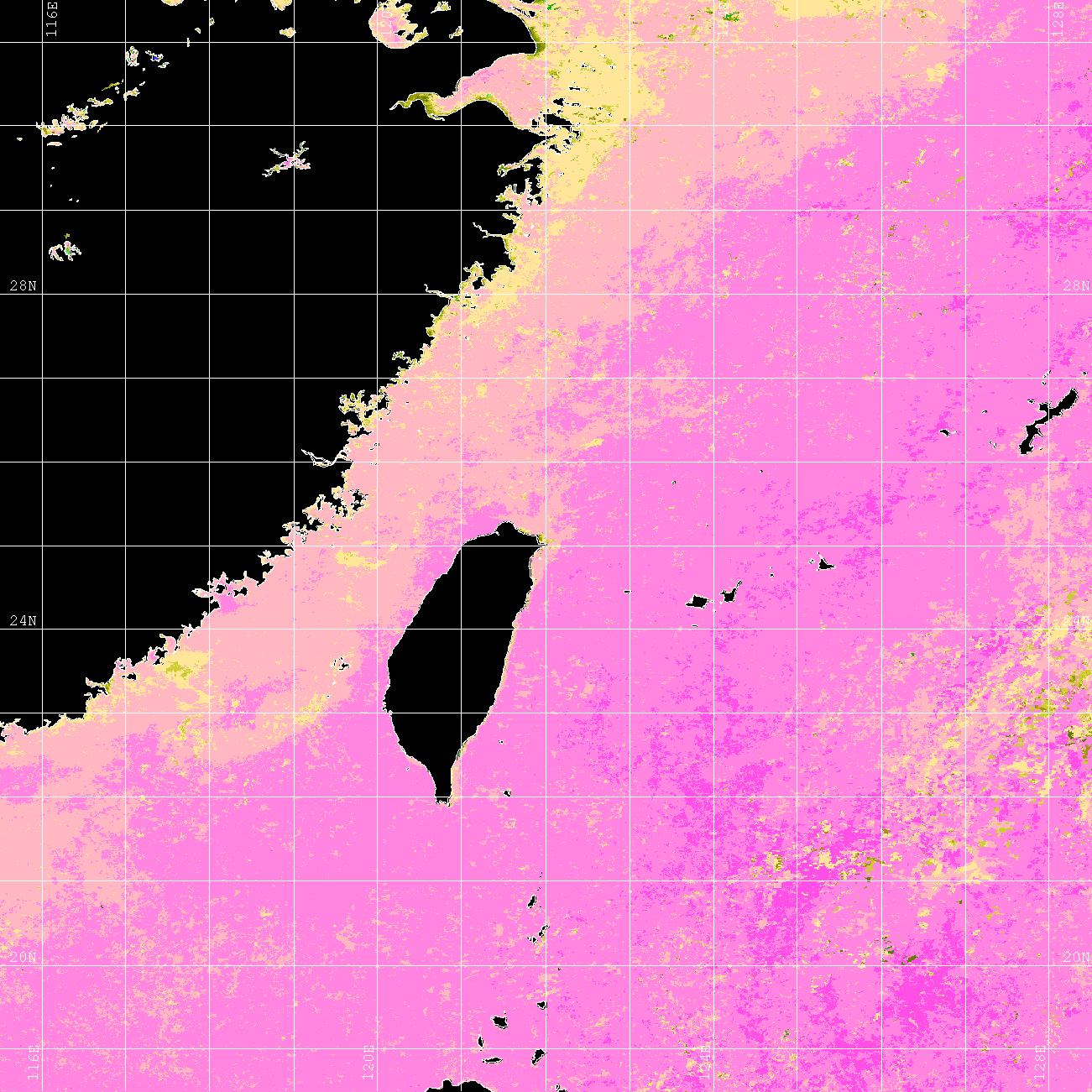

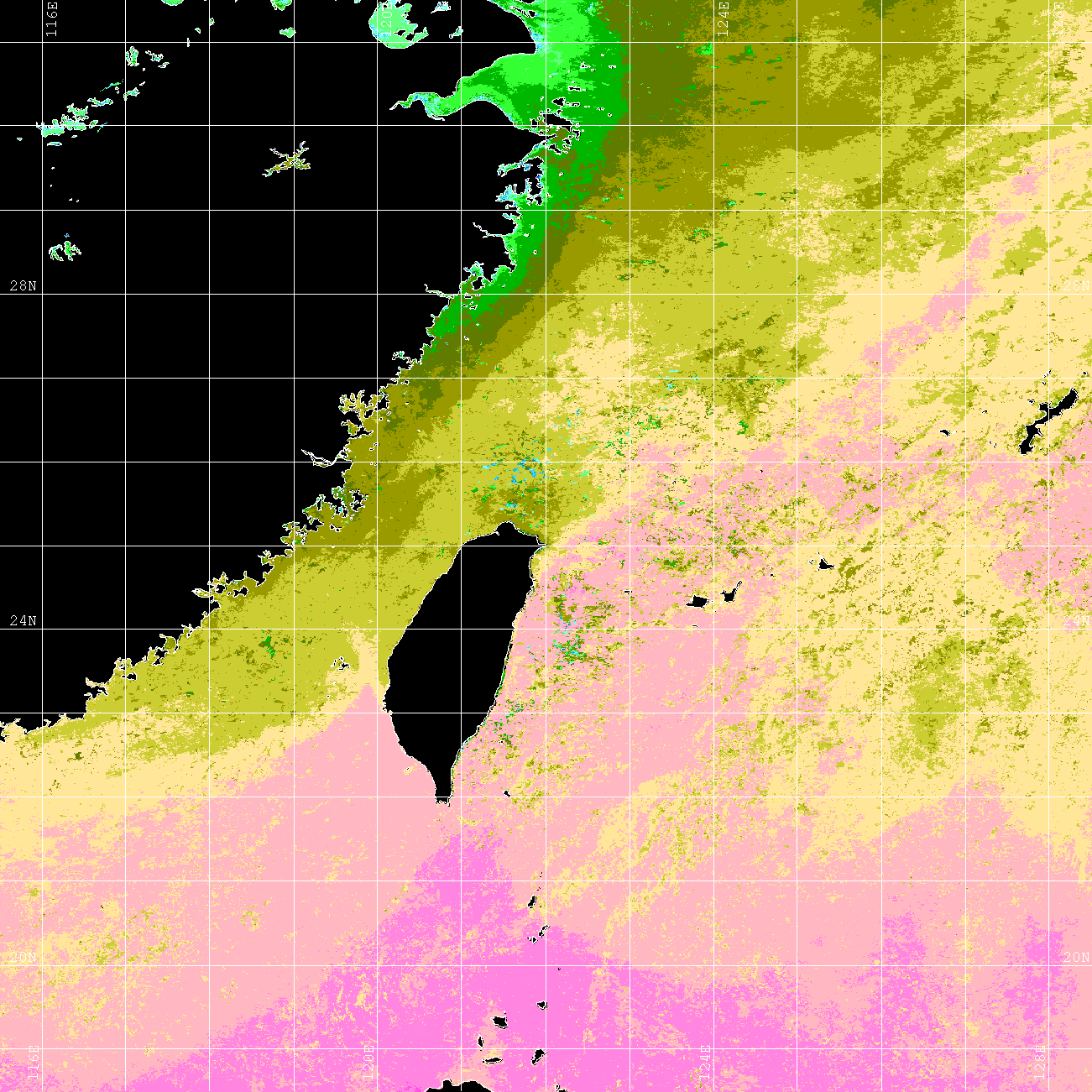

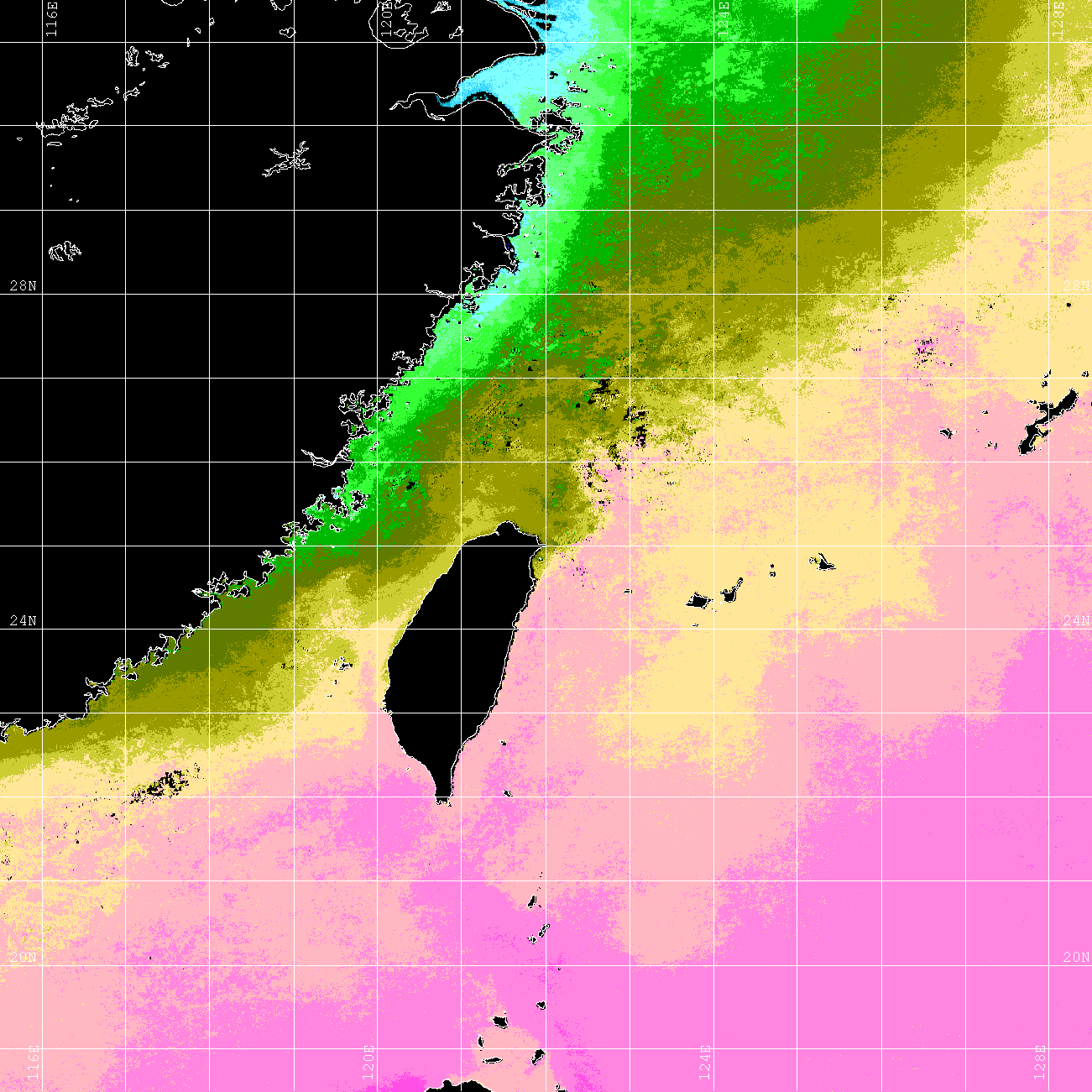

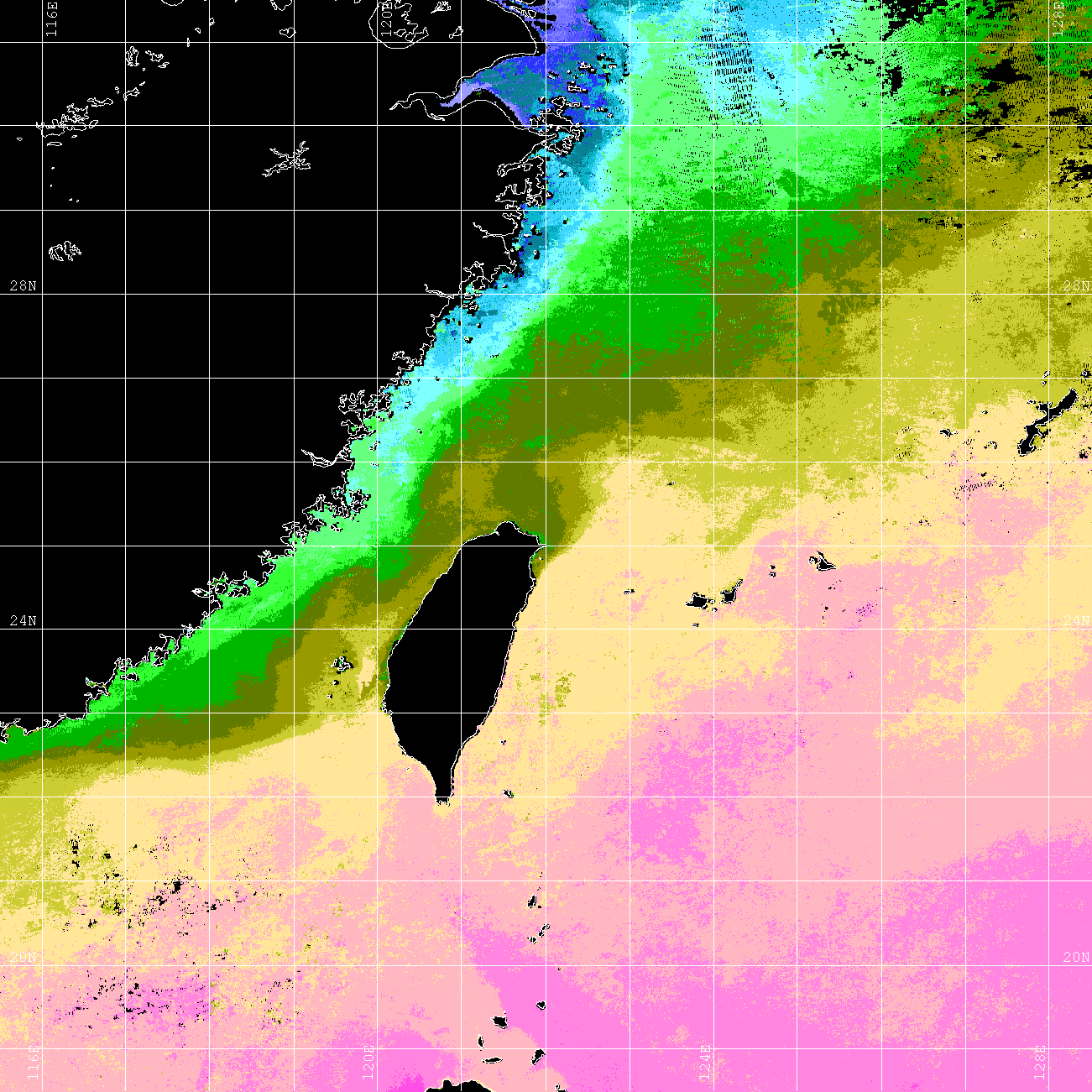


l) December

k) November

j) October

i) September

l)

**Figure S2. Seasonal sea surface temperature (SST) distribution for 2018.** (a-l) Large-scale SST distribution for 12 months (January–December 2018) for the cold waters off northwestern Taiwan and warm waters off southeastern Taiwan. The contour line of SST < 24 °C was located in the region off southeastern Taiwan in (a) cool January, moved northward to the regions off northwestern Taiwan in (e) warm May, became spotty in the study area in (h) hot August, and gradually moved southward to the regions off southeast Taiwan until (l) cool December. Validation and data source: the Visible Infrared Imaging Radiometer Suite (VIIRS) onboard the Suomi National Polar-Orbiting Partnership (S-NPP) satellite^1^. The color bar displays values of SST. This image was created by TeraScan (Version 4.1.2), SeaSpace.

Tu Q, Pan D, Hao Z (2015) Validation of S-NPP VIIRS sea surface temperature retrieved from NAVO. Remote Sens 7:17234–17245. doi:10.3390/rs71215881

**Table S1. Seawater sampling date (local time, year/month/day), latitude (°N), longitude (°E), depth (m), temperature (°C), salinity, and ^137^Cs and ^134^Cs activity concentrations (Bq**∙**m^-3^) in this study.**

| Sampling date | Lat. (°N) | Long. (°E) | Sample depth (m) | Temp. (°C) | Salinity | Sigma_Theta (kg·m^-3^) | ^137^CS (Bq·m^-3^) | ^137^CS Error (Bq·m^-3^) | ^134^CS (Bq·m^-3^) |
| --- | --- | --- | --- | --- | --- | --- | --- | --- | --- |
| 2018/02/25 | 21.8978 | 120.7493 | 0 | 24.2 | 34.7 | 23.24 | 1.0 | ±0.2 | - |
| 2018/02/25 | 21.9056 | 120.8141 | 0 | 25.2 | 34.6 | 22.86 | 1.0 | ±0.2 | - |
| 2018/02/25 | 21.9553 | 120.7624 | 0 | 25.1 | 34.5 | 22.82 | 1.3 | ±0.2 | - |
| 2018/01/03 | 25.4131 | 122.1988 | 3.5 | 20.4 | 34.6 | 24.25 | 1.4 | ±0.2 | - |
| 2018/01/03 | 25.4144 | 122.2005 | 200 | 14.5 | 34.6 | 26.53 | 1.4 | ±0.3 | - |
| 2018/02/09 | 26.1653 | 119.9529 | 0 | 9.7 | 29.1 | 22.31 | 1.8 | ±0.3 | - |
| 2018/03/11 | 24.4049 | 118.3947 | 0 | 14.8 | 31 | 22.84 | 1.1 | ±0.1 | - |
| 2018/03/11 | 24.3927 | 118.4221 | 0 | 14.8 | 31.6 | 23.30 | 1.2 | ±0.3 | - |
| 2018/03/11 | 24.3791 | 118.4486 | 0 | 14.6 | 31.7 | 23.42 | 1.2 | ±0.3 | - |
| 2018/03/10 | 26.1600 | 120.0075 | 0 | 12.6 | 30.6 | 22.97 | 1.0 | ±0.2 | - |
| 2018/03/10 | 26.1592 | 119.9995 | 0 | 12.6 | 30.6 | 22.97 | 1.3 | ±0.2 | - |
| 2018/03/10 | 26.1656 | 119.9841 | 0 | 12.7 | 30.8 | 23.11 | 1.0 | ±0.3 | - |
| 2018/03/15 | 22.6355 | 120.1670 | 0 | 26.4 | 34 | 22.05 | 0.9 | ±0.2 | - |
| 2018/03/10 | 23.7519 | 120.1784 | 0 | 18.6 | 29.2 | 20.63 | 1.1 | ±0.2 | - |
| 2018/03/14 | 24.3415 | 121.7858 | 220 | 17.7 | 34.4 | 25.73 | 1.1 | ±0.3 | - |
| 2018/03/14 | 25.1857 | 121.7961 | 2.5 | 18.9 | 34.2 | 24.36 | 1.5 | ±0.2 | - |
| 2018/03/18 | 25.1338 | 121.9933 | 2.5 | 20.9 | 34.4 | 23.93 | 1.4 | ±0.2 | - |
| 2018/03/20 | 22.6340 | 120.2379 | 0 | 26.6 | 34.0 | 22.00 | 0.9 | ±0.2 | - |
| 2018/03/20 | 22.5649 | 120.2029 | 0 | 25.7 | 33.7 | 22.07 | 1.3 | ±0.2 | - |
| 2018/03/22 | 25.1573 | 121.8464 | 2.5 | 22.3 | 34.3 | 23.50 | 1.0 | ±0.2 | - |
| 2018/03/31 | 22.2321 | 120.3999 | 0 | 26.24 | 34.3 | 22.32 | 1.1 | ±0.2 | - |
| 2018/04/02 | 25.4182 | 122.2057 | 0 | 21.3 | 34.5 | 23.92 | 1.6 | ±0.2 | - |
| 2018/04/02 | 25.4463 | 122.0796 | 0 | 21.4 | 34.5 | 23.89 | 1.4 | ±0.2 | - |
| 2018/04/02 | 25.2479 | 122.3170 | 0 | 22.6 | 34.3 | 23.41 | 1.4 | ±0.2 | - |
| 2018/04/02 | 25.4182 | 122.2057 | 200 | 15.1 | 34.6 | 26.40 | 2.0 | ±0.3 | - |
| 2018/04/02 | 25.4463 | 122.0796 | 200 | 15.3 | 34.6 | 26.36 | 2.0 | ±0.1 | - |
| 2018/04/03 | 22.3662 | 120.2986 | 0 | 25.52 | 34.52 | 22.71 | 1.0 | ±0.3 | - |
| 2018/04/27 | 22.6770 | 120.1654 | 0 | 26.7 | 34.3 | 22.18 | 1.6 | ±0.3 | - |
| 2018/05/05 | 26.1804 | 119.9658 | 0 | 22.4 | 32.3 | 21.97 | 1.2 | ±0.2 | - |
| 2018/05/05 | 26.1596 | 119.9929 | 0 | 22 | 32.5 | 22.23 | 1.6 | ±0.3 | - |
| 2018/05/05 | 26.1518 | 120.0217 | 0 | 22.1 | 32.7 | 22.35 | 1.2 | ±0.3 | - |
| 2018/05/14 | 21.8978 | 120.7493 | 0 | 28 | 34.099 | 21.61 | 1.1 | ±0.2 | - |
| 2018/05/14 | 21.9056 | 120.8141 | 0 | 29.6 | 34.1 | 21.09 | 1.0 | ±0.2 | - |
| 2018/05/14 | 21.9553 | 120.7624 | 0 | 29.2 | 33.8 | 21.00 | 1.1 | ±0.2 | - |
| 2018/05/18 | 24.4037 | 118.3915 | 0 | 25.8 | 33.4 | 21.79 | 1.9 | ±0.3 | - |
| 2018/05/18 | 24.3887 | 118.4191 | 0 | 26.3 | 33.4 | 21.64 | 1.3 | ±0.2 | - |
| 2018/05/18 | 24.3741 | 118.4428 | 0 | 26.3 | 33.4 | 21.64 | 1.2 | ±0.3 | - |
| 2018/05/29 | 22.6311 | 120.2276 | 3 | 30.205 | 33.067 | 20.14 | 1.5 | ±0.3 | - |
| 2018/06/12 | 22.6489 | 120.1852 | 0 | 30 | 32.8 | 20.00 | 1.9 | ±0.3 | - |
| 2018/06/29 | 22.6441 | 120.1779 | 0.5 | 29.9 | 32.7 | 19.96 | 0.9 | ±0.1 | - |
| 2018/05/27 | 25.1545 | 121.8403 | 1 | 25 | 32 | 21.00 | 1.8 | ±0.3 | - |
| 2018/06/08 | 25.1663 | 121.8343 | 2 | 27.2 | 34.1 | 21.88 | 1.8 | ±0.3 | - |
| 2018/07/04 | 25.2525 | 121.7357 | 1 | 28.3 | 33.9 | 21.37 | 1.4 | ±0.2 | - |
| 2018/07/08 | 25.6867 | 121.3795 | 5 | 28.7 | 34.0 | 21.32 | 1.1 | ±0.2 | - |
| 2018/07/08 | 25.5498 | 121.8074 | 5 | 28.2 | 34.0 | 21.49 | 1.7 | ±0.3 | - |
| 2018/07/25 | 22.6281 | 120.1981 | 1 | 29.7 | 32.5 | 19.89 | 1.3 | ±0.2 | - |
| 2018/08/12 | 26.1519 | 120.0210 | 0 | 29.4 | 32.8 | 20.20 | 1.2 | ±0.2 | - |
| 2018/08/12 | 26.1596 | 119.9923 | 0 | 29.6 | 32.7 | 20.06 | 1.9 | ±0.2 | - |
| 2018/08/12 | 26.1805 | 119.9660 | 0 | 28.8 | 32.1 | 19.89 | 1.2 | ±0.2 | - |
| 2018/08/26 | 24.4056 | 118.3969 | 0 | 29.2 | 32.0 | 19.68 | 1.8 | ±0.2 | - |
| 2018/08/26 | 24.3890 | 118.4220 | 0 | 29.4 | 32.2 | 19.76 | 1.6 | ±0.2 | - |
| 2018/08/26 | 24.3724 | 118.4429 | 0 | 29.2 | 32.4 | 19.98 | 1.5 | ±0.2 | - |
| 2018/08/17 | 22.5022 | 120.1730 | 5 | 29.78 | 33.32 | 20.48 | 1.1 | ±0.2 | - |
| 2018/08/17 | 22.5022 | 120.1730 | 200 | 15.43 | 34.59 | 26.32 | 2.0 | ±0.3 | - |
| 2018/08/18 | 21.8772 | 117.4076 | 5 | 29.18 | 33.18 | 20.57 | 0.8 | ±0.2 | - |
| 2018/08/18 | 21.8772 | 117.4076 | 200 | 16.85 | 34.63 | 26.02 | 2.0 | ±0.2 | - |
| 2018/08/12 | 25.1613 | 121.8440 | 2 | 27.5 | 33.2 | 21.12 | 1.5 | ±0.3 | - |
| 2018/09/07 | 21.8978 | 120.7493 | 0 | 29.2 | 31.613 | 19.40 | 1.0 | ±0.2 | - |
| 2018/09/07 | 21.9056 | 120.8141 | 0 | 29.3 | 32.189 | 19.79 | 0.8 | ±0.2 | - |
| 2018/09/07 | 21.9553 | 120.7624 | 0 | 30.1 | 32.401 | 19.68 | 1.1 | ±0.2 | - |
| 2018/09/08 | 22.5124 | 120.2020 | 5 | 29.2177 | 32.944 | 20.39 | 0.9 | ±0.2 | - |
| 2018/09/08 | 22.5124 | 120.2020 | 178 | 16.216 | 34.5755 | 26.03 | 1.9 | ±0.2 | - |
| 2018/10/15 | 26.1504 | 120.0203 | 0 | 22.7 | 29.1 | 19.51 | 1.1 | ±0.2 | - |
| 2018/10/15 | 26.1572 | 119.9895 | 0 | 23.3 | 29.1 | 19.34 | 1.1 | ±0.2 | - |
| 2018/10/15 | 26.1744 | 119.9683 | 0 | 23.3 | 29 | 19.27 | 1.7 | ±0.3 | - |
| 2018/09/18 | 22.8606 | 121.2026 | 0 | 29.4 | 31.6 | 19.32 | 1.2 | ±0.2 | - |
| 2018/09/22 | 25.1682 | 121.8397 | 2 | 27.5 | 33.1 | 21.05 | 1.8 | ±0.2 | - |
| 2018/10/18 | 22.6305 | 120.1952 | 0 | 28 | 34.6 | 21.98 | 0.9 | ±0.2 | - |
| 2018/10/19 | 24.4049 | 118.3947 | 0 | 23.5 | 31.9 | 21.36 | 1.0 | ±0.2 | - |
| 2018/10/19 | 24.3927 | 118.4221 | 0 | 23.4 | 32 | 21.47 | 1.4 | ±0.3 | - |
| 2018/10/19 | 24.3791 | 118.4486 | 0 | 23.2 | 32 | 21.52 | 1.0 | ±0.2 | - |
| 2018/10/23 | 25.2124 | 122.0207 | 2 | 24.4 | 33.6 | 22.37 | 1.8 | ±0.2 | - |
| 2018/11/06 | 22.7548 | 120.1236 | 1 | 27.6 | 33 | 20.94 | 1.0 | ±0.2 | - |
| 2018/11/07 | 21.8978 | 120.7493 | 0 | 25.7 | 34.3 | 22.49 | 1.3 | ±0.2 | - |
| 2018/11/07 | 21.9056 | 120.8141 | 0 | 26.8 | 34.5 | 22.29 | 1.1 | ±0.3 | - |
| 2018/11/07 | 21.9553 | 120.7624 | 0 | 26.3 | 34.7 | 22.60 | 1.2 | ±0.2 | - |
| 2018/11/10 | 23.1336 | 120.0567 | 0 | 27.4 | 32.9 | 20.93 | 1.0 | ±0.2 | - |
| 2018/11/13 | 22.6537 | 120.1623 | 1 | 27.1 | 33.1 | 21.17 | 1.4 | ±0.2 | - |
| 2018/11/07 | 25.2260 | 121.7098 | 2 | 22.9 | 33.4 | 22.66 | 1.7 | ±0.2 | - |
| 2018/11/26 | 25.1491 | 121.8119 | 2 | 22 | 33.7 | 23.13 | 1.5 | ±0.4 | - |
| 2018/03/21 | 22.6175 | 120.2682 | 1 | 26.3 | 32.3 | 20.83 | 1.2 | ±0.2 | - |
| 2018/03/14 | 25.1446 | 121.7915 | 1 | 20.8 | 33.2 | 23.08 | 1.4 | ±0.2 | - |
| 2018/03/20 | 23.4507 | 120.1376 | 1 | 24.7 | 32.3 | 21.32 | 0.9 | ±0.2 | - |
| 2018/03/13 | 23.9762 | 121.6198 | 1 | 24.8 | 33.1 | 21.88 | 1.4 | ±0.3 | - |
| 2018/03/13 | 23.1070 | 121.3893 | 1 | 25.6 | 33.9 | 22.23 | 1.6 | ±0.3 | - |
| 2018/03/14 | 22.3383 | 120.8989 | 1 | 25 | 27.2 | 17.46 | 1.4 | ±0.3 | - |
| 2018/03/14 | 24.5820 | 121.8684 | 1 | 22.3 | 30.8 | 20.89 | 0.9 | ±0.2 | - |
| 2018/03/19 | 24.8463 | 120.9237 | 1 | 24.4 | 33.2 | 22.07 | 1.4 | ±0.3 | - |
| 2018/03/19 | 23.9721 | 120.3238 | 1 | 26.8 | 32.5 | 20.82 | 1.1 | ±0.2 | - |
| 2018/04/11 | 23.3848 | 120.1511 | 1 | 26.4 | 33.6 | 21.76 | 0.7 | ±0.2 | - |
| 2018/04/10 | 24.8463 | 120.9237 | 1 | 25.1 | 30.2 | 19.64 | 1.1 | ±0.1 | - |
| 2018/04/10 | 23.9721 | 120.3238 | 1 | 28.9 | 33.6 | 20.96 | 1.3 | ±0.3 | - |
| 2018/04/15 | 23.9762 | 121.6198 | 1 | 23.9 | 33.2 | 22.22 | 1.0 | ±0.2 | - |
| 2018/04/15 | 22.3383 | 120.8989 | 1 | 25.1 | 33.3 | 21.94 | 1.4 | ±0.2 | - |
| 2018/04/15 | 23.1070 | 121.3893 | 1 | 26 | 33.3 | 21.66 | 1.3 | ±0.1 | - |
| 2018/04/18 | 25.1446 | 121.7915 | 1 | 23.2 | 32.7 | 22.05 | 1.0 | ±0.2 | - |
| 2018/04/20 | 24.5820 | 121.8684 | 1 | 24.7 | 32.4 | 21.39 | 1.5 | ±0.1 | - |
| 2018/05/15 | 22.6175 | 120.2682 | 1 | 30.6 | 31.5 | 18.85 | 2.0 | ±0.4 | - |
| 2018/07/06 | 22.6175 | 120.2682 | 1 | 27.5 | 30.5 | 19.13 | 0.6 | ±0.1 | - |
| 2018/07/10 | 24.8463 | 120.9237 | 1 | 30.2 | 31.5 | 18.99 | 0.9 | ±0.2 | - |
| 2018/07/10 | 23.9721 | 120.3238 | 1 | 34.9 | 32.5 | 18.06 | 1.0 | ±0.2 | - |
| 2018/07/11 | 23.4507 | 120.1376 | 1 | 31.5 | 26.5 | 14.91 | 0.7 | ±0.2 | - |
| 2018/07/18 | 22.7910 | 121.1920 | 1 | 29.1 | 30.3 | 18.48 | 1.2 | ±0.2 | - |
| 2018/07/19 | 23.9762 | 121.6198 | 1 | 30.3 | 30.3 | 18.08 | 1.1 | ±0.2 | - |
| 2018/07/19 | 23.1070 | 121.3893 | 1 | 29.4 | 31.5 | 19.25 | 1.3 | ±0.2 | - |
| 2018/07/31 | 25.1446 | 121.7915 | 1 | 31 | 32.4 | 19.37 | 1.4 | ±0.2 | - |
| 2018/08/01 | 24.5820 | 121.8684 | 1 | 31.7 | 32 | 18.84 | 0.7 | ±0.1 | - |
| 2018/10/04 | 23.9762 | 121.6198 | 1 | 27.4 | 32.6 | 20.71 | 0.8 | ±0.2 | - |
| 2018/10/04 | 22.7910 | 121.1920 | 1 | 27.2 | 32.3 | 20.55 | 0.7 | ±0.2 | - |
| 2018/10/04 | 23.1070 | 121.3893 | 1 | 27.3 | 32.5 | 20.67 | 0.8 | ±0.2 | - |
| 2018/10/16 | 24.8463 | 120.9237 | 1 | 29.1 | 24.5 | 14.23 | 0.9 | ±0.1 | - |
| 2018/10/17 | 23.9721 | 120.3238 | 1 | 24.7 | 32.7 | 21.61 | 1.3 | ±0.2 | - |
| 2018/10/17 | 23.4507 | 120.1376 | 1 | 27.4 | 31.2 | 19.68 | 0.8 | ±0.2 | - |
| 2018/10/31 | 24.5820 | 121.8684 | 1 | 22.9 | 31.5 | 21.24 | 1.0 | ±0.2 | - |
| 2018/11/02 | 22.6175 | 120.2682 | 1 | 27.9 | 31.8 | 19.96 | 1.0 | ±0.2 | - |
| 2018/11/07 | 25.1446 | 121.7915 | 1 | 25.3 | 32.6 | 21.36 | 2.0 | ±0.3 | - |
| 2019/02/14 | 22.0043222 | 121.5810583 | 1 | 26.6 | 33.9 | 21.92 | 1.1 | ±0.2 | - |
| 2019/02/22 | 21.89775 | 120.7493056 | 1 | 24.5 | 34.6 | 23.08 | 1.3 | ±0.2 | - |
| 2019/02/22 | 21.9056389 | 120.8140833 | 1 | 26.3 | 34 | 22.09 | 1.2 | ±0.2 | - |
| 2019/02/22 | 21.95525 | 120.7624444 | 1 | 25.7 | 34.4 | 22.57 | 1.2 | ±0.2 | - |
| 2019/02/21 | 22.0572389 | 119.7995278 | 3 | 26.4 | 34.6 | 22.51 | 1.1 | ±0.2 | - |
| 2019/02/21 | 22.0572389 | 119.7995278 | 50 | 25.9251 | 34.5149 | 22.79 | 1.6 | ±0.3 | - |
| 2019/02/21 | 22.0572389 | 119.7995278 | 125 | 22.1388 | 34.9147 | 24.53 | 1.3 | ±0.2 | - |
| 2019/02/21 | 22.0572389 | 119.7995278 | 200 | 17.0057 | 34.7171 | 26.05 | 2.2 | ±0.2 | - |
| 2019/02/22 | 22.4173611 | 120.0459389 | 5 | 26.4 | 34.2 | 22.22 | 1.0 | ±0.2 | - |
| 2019/02/24 | 25.2007833 | 121.8238972 | 5 | 20.65 | 34.47 | 24.09 | 1.1 | ±0.2 | - |
| 2019/02/24 | 25.2007833 | 121.8238972 | 50 | 20.03 | 34.5 | 24.47 | 1.3 | ±0.2 | - |
| 2019/02/24 | 25.2007833 | 121.8238972 | 100 | 18.89 | 34.48 | 24.97 | 1.3 | ±0.2 | - |
| 2019/02/27 | 25.4117639 | 121.5455167 | 5 | 21.49 | 34.49 | 23.88 | 0.9 | ±0.2 | - |
| 2019/02/27 | 25.4117639 | 121.5455167 | 50 | 21.32 | 34.487 | 24.12 | 0.9 | ±0.2 | - |
| 2019/02/27 | 25.4117639 | 121.5455167 | 105 | 20.19 | 34.47 | 24.65 | 1.0 | ±0.2 | - |
| 2019/03/04 | 10.3786111 | 114.365 | 1 | 26 | 31.5 | 20.33 | 1.1 | ±0.2 | - |
| 2019/03/27 | 25.4178333 | 122.2069972 | 1 | 25.422 | 34.611 | 22.81 | 0.9 | ±0.1 | - |
| 2019/03/28 | 25.4178333 | 122.2069972 | 50 | 22.661 | 34.7243 | 23.92 | 1.1 | ±0.2 | - |
| 2019/03/27 | 25.4178333 | 122.2069972 | 125 | 18.7167 | 34.7807 | 25.35 | 1.7 | ±0.2 | - |
| 2019/03/27 | 25.4178333 | 122.2069972 | 200 | 15.8307 | 34.6097 | 26.24 | 1.9 | ±0.2 | - |
| 2019/03/27 | 25.7508306 | 121.2578333 | 1 | 22.9812 | 34.518 | 23.46 | 1.1 | ±0.1 | - |
| 2019/03/27 | 25.7508306 | 121.2578333 | 50 | 22.0039 | 34.4857 | 23.93 | 1.1 | ±0.2 | - |
| 2019/03/27 | 25.7508306 | 121.2578333 | 75 | 21.8026 | 34.4759 | 24.08 | 1.3 | ±0.2 | - |
| 2019/04/20 | 25.1741639 | 121.9206667 | 1 | 21.785 | 34.58 | 23.85 | 1.2 | ±0.1 | - |
| 2019/04/20 | 25.1741639 | 121.9206667 | 50 | 19.5799 | 34.5986 | 24.66 | 1.2 | ±0.2 | - |
| 2019/04/20 | 25.1741639 | 121.9206667 | 125 | 19.379 | 34.6038 | 25.04 | 1.9 | ±0.4 | - |
| 2019/04/20 | 25.1741639 | 121.9206667 | 200 | 17.9148 | 34.6316 | 25.76 | 1.5 | ±0.2 | - |
| 2019/04/26 | 25.3961139 | 121.3569333 | 1 | 26.32 | 33.822 | 21.95 | 1.2 | ±0.2 | - |
| 2019/05/01 | 22.9914639 | 119.9818 | 1 | 27.9165 | 34.416 | 21.88 | 1.1 | ±0.2 | - |
| 2019/05/01 | 22.9914639 | 119.9818 | 50 | 27.5578 | 34.4288 | 22.21 | 1.8 | ±0.3 | - |
| 2019/05/01 | 22.9914639 | 119.9818 | 75 | 27.3772 | 34.4207 | 22.37 | 1.3 | ±0.2 | - |
| 2019/05/01 | 22.9914639 | 119.9818 | 100 | 24.5405 | 34.6207 | 23.50 | 1.0 | ±0.2 | - |
| 2019/05/09 | 21.89775 | 120.7493056 | 1 | 27.8 | 34.575 | 22.03 | 0.9 | ±0.2 | - |
| 2019/05/09 | 21.9056389 | 120.8140833 | 1 | 28 | 34.591 | 21.98 | 1.0 | ±0.2 | - |
| 2019/05/09 | 21.95525 | 120.7624444 | 1 | 27.7 | 34.595 | 22.08 | 1.1 | ±0.1 | - |
| 2019/05/25 | 23.0765556 | 119.9486806 | 1 | 28.0604 | 34.2572 | 21.71 | 1.4 | ±0.2 | - |
| 2019/05/25 | 23.0765556 | 119.9486806 | 50 | 26.4891 | 34.4857 | 22.59 | 1.0 | ±0.2 | - |
| 2019/05/25 | 23.0765556 | 119.9486806 | 75 | 26.0583 | 34.5563 | 22.89 | 1.3 | ±0.2 | - |
| 2019/05/25 | 23.0765556 | 119.9486806 | 100 | 24.905 | 34.5608 | 23.35 | 1.1 | ±0.2 | - |
| 2019/06/03 | 21.9394972 | 122.6126639 | 1 | 29.4 | 34.52 | 21.46 | 1.0 | ±0.2 | - |
| 2019/06/03 | 21.9394972 | 122.6126639 | 50 | 26.92 | 34.72 | 22.63 | 0.9 | ±0.3 | - |
| 2019/06/03 | 21.9394972 | 122.6126639 | 125 | 24.75 | 34.78 | 23.66 | 1.1 | ±0.2 | - |
| 2019/06/03 | 21.9394972 | 122.6126639 | 200 | 22.61 | 34.93 | 24.73 | 1.4 | ±0.2 | - |
| 2019/06/19 | 24.8310972 | 121.9642306 | 1 | 30 | 34.4 | 21.17 | 1.3 | ±0.2 | - |
| 2019/08/15 | 23.6200806 | 119.8899472 | 1 | 33.37 | 29.4 | 16.37 | 1.3 | ±0.3 | - |
| 2019/08/18 | 22 | 119.6799 | 5 | 29.34 | 34.08 | 21.18 | 1.1 | ±0.2 | - |
| 2019/08/18 | 22 | 119.6799 | 110 | 22.78 | 34.71 | 24.13 | 1.5 | ±0.2 | - |
| 2019/08/18 | 22 | 119.6799 | 300 | 12.45 | 34.43 | 27.27 | 2.2 | ±0.4 | - |
| 2019/08/23 | 21.89775 | 120.7493056 | 1 | 27.8 | 33.18 | 21.01 | 1.1 | ±0.2 | - |
| 2019/08/23 | 21.9056389 | 120.8140833 | 1 | 29.6 | 33.52 | 20.67 | 1.4 | ±0.2 | - |
| 2019/08/23 | 21.95525 | 120.7624444 | 1 | 29.6 | 33.18 | 20.42 | 1.5 | ±0.2 | - |
| 2019/08/21 | 23.747 | 119.7203667 | 1 | 27 | 33.9 | 21.79 | 1.3 | ±0.2 | - |
| 2019/08/21 | 23.7292 | 119.6709167 | 1 | 26.9 | 33.9 | 21.82 | 1.4 | ±0.2 | - |
| 2019/08/21 | 23.6892167 | 119.6165 | 1 | 27.3 | 33.8 | 21.62 | 1.5 | ±0.3 | - |
| 2019/09/28 | 23.0765556 | 119.9486806 | 1 | 28.25 | 33.634 | 21.20 | 1.4 | ±0.2 | - |
| 2019/09/28 | 23.0765556 | 119.9486806 | 50 | 27.9 | 33.839 | 21.67 | 1.1 | ±0.1 | - |
| 2019/09/28 | 23.7292 | 119.6709167 | 1 | 26.5 | 34.2 | 22.17 | 1.2 | ±0.2 | - |
| 2019/09/28 | 23.6892167 | 119.6165 | 1 | 26.3 | 34.2 | 22.23 | 1.5 | ±0.1 | - |
| 2019/10/05 | 25.7508306 | 121.2578333 | 1 | 26.7661 | 33.0213 | 21.22 | 1.6 | ±0.2 | - |
| 2019/10/05 | 25.7508306 | 121.2578333 | 50 | 25.9798 | 33.8812 | 22.31 | 1.4 | ±0.2 | - |
| 2019/10/05 | 25.3961139 | 121.3569333 | 1 | 26.8314 | 32.6632 | 20.93 | 1.2 | ±0.2 | - |
| 2019/10/05 | 25.3961139 | 121.3569333 | 50 | 26.295 | 33.7192 | 22.09 | 1.6 | ±0.2 | - |
| 2019/10/07 | 21.8954667 | 122.5815139 | 1 | 27.948 | 34.341 | 21.81 | 1.2 | ±0.1 | - |
| 2019/10/07 | 21.9 | 122.5754833 | 50 | 22.244 | 34.894 | 24.16 | 1.2 | ±0.2 | - |
| 2019/10/07 | 21.9057833 | 122.59385 | 125 | 21.1 | 34.92 | 24.82 | 1.5 | ±0.1 | - |
| 2019/10/07 | 21.9057833 | 122.59385 | 200 | 18.327 | 34.842 | 25.82 | 2.0 | ±0.2 | - |
| 2019/10/07 | 21.8954667 | 122.5815139 | 400 | 12.481 | 34.358 | 27.65 | 2.1 | ±0.2 | - |
| 2019/10/07 | 21.8954667 | 122.5815139 | 600 | 7.412 | 34.275 | 29.39 | 0.9 | ±0.2 | - |
| 2019/10/07 | 21.8943 | 122.5814139 | 800 | 5.756 | 34.386 | 30.63 | 0.7 | ±0.2 | - |
| 2019/10/07 | 21.8943 | 122.5814139 | 1000 | 4.73 | 34.453 | 31.73 | 0.8 | ±0.1 | - |
| 2019/10/11 | 23.6200806 | 119.8899472 | 1 | 28.2 | 34 | 21.48 | 1.1 | ±0.2 | - |
| 2019/10/11 | 23.6200806 | 119.8899472 | 50 | 25 | 34.1 | 22.77 | 1.4 | ±0.2 | - |
| 2019/01/07 | 24.8474528 | 120.9244972 | 1 | 20.2 | 31.2 | 21.74 | 1.4 | ±0.2 | - |
| 2019/01/08 | 23.9719694 | 120.3238806 | 1 | 20.5 | 31.8 | 22.11 | 1.4 | ±0.2 | - |
| 2019/01/09 | 23.4530389 | 120.1381556 | 1 | 20.5 | 31.7 | 22.04 | 1.6 | ±0.2 | - |
| 2019/01/08 | 22.6175333 | 120.2681806 | 1 | 26.6 | 32 | 20.52 | 0.9 | ±0.2 | - |
| 2019/01/15 | 23.9811667 | 121.6243611 | 1 | 24.4 | 32.1 | 21.26 | 1.4 | ±0.2 | - |
| 2019/01/15 | 23.1594722 | 121.4030278 | 1 | 24.2 | 31.1 | 20.57 | 1.1 | ±0.2 | - |
| 2019/01/15 | 22.7908056 | 121.19225 | 1 | 25.5 | 33 | 21.59 | 1.1 | ±0.2 | - |
| 2019/01/18 | 24.5819778 | 121.86835 | 1 | 15.9 | 31.8 | 23.22 | 1.0 | ±0.2 | - |
| 2019/01/25 | 25.1445778 | 121.7914778 | 1 | 20.3 | 31.2 | 21.72 | 1.3 | ±0.2 | - |
| 2019/01/15 | 10.5 | 114.2999972 | 1 | 28.4 | 30.4 | 18.77 | 1.5 | ±0.2 | - |
| 2019/01/15 | 10.5 | 114.5 | 1 | 28.4 | 30.8 | 19.07 | 1.1 | ±0.2 | - |
| 2019/01/15 | 10.4000028 | 114.2000028 | 1 | 28.5 | 30.8 | 19.04 | 1.1 | ±0.2 | - |
| 2019/02/12 | 21.8000056 | 120.7000028 | 1 | 25 | 32.4 | 21.30 | 0.8 | ±0.2 | - |
| 2019/02/12 | 21.96 | 120.59 | 1 | 25 | 32.4 | 21.30 | 0.8 | ±0.2 | - |
| 2019/02/12 | 21.8599972 | 120.93 | 1 | 25 | 32.1 | 21.08 | 1.2 | ±0.2 | - |
| 2019/01/31 | 23.9 | 119.5833333 | 1 | 23.8 | 32.1 | 21.43 | 0.9 | ±0.2 | - |
| 2019/01/31 | 23.7166667 | 119.3166667 | 1 | 23.6 | 32.4 | 21.71 | 1.3 | ±0.1 | - |
| 2019/01/31 | 23.3333333 | 119.6666667 | 1 | 24.2 | 32.4 | 21.54 | 1.2 | ±0.1 | - |
| 2019/01/25 | 26.4666667 | 120.6166667 | 1 | 13.3 | 28.7 | 21.39 | 1.7 | ±0.2 | - |
| 2019/01/25 | 26.2833333 | 120.6166667 | 1 | 16.5 | 31.1 | 22.56 | 1.3 | ±0.2 | - |
| 2019/01/25 | 26.3 | 120.4 | 1 | 16.3 | 30.8 | 22.37 | 0.9 | ±0.3 | - |
| 2019/02/19 | 26.04 | 119.87 | 1 | 14.2 | 27.7 | 20.46 | 0.9 | ±0.2 | - |
| 2019/02/19 | 26.06 | 120 | 1 | 14.3 | 27.7 | 20.44 | 1.2 | ±0.1 | - |
| 2019/02/19 | 26.14 | 120.08 | 1 | 14.1 | 27.7 | 20.47 | 1.0 | ±0.1 | - |
| 2019/02/12 | 24.4 | 118.5 | 1 | 18 | 28.2 | 20.02 | 0.9 | ±0.2 | - |
| 2019/02/15 | 24.37 | 118.45 | 1 | 15 | 28.6 | 20.98 | 1.4 | ±0.2 | - |
| 2019/02/18 | 24.35 | 118.32 | 1 | 15 | 28.8 | 21.13 | 1.1 | ±0.2 | - |
| 2019/04/08 | 24.8474528 | 120.9244972 | 1 | 26.4 | 30.6 | 19.55 | 1.0 | ±0.2 | - |
| 2019/04/09 | 23.9719694 | 120.3238806 | 1 | 27.8 | 31.2 | 19.55 | 0.9 | ±0.1 | - |
| 2019/04/09 | 23.4530389 | 120.1381556 | 1 | 26.8 | 31.8 | 20.31 | 1.4 | ±0.2 | - |
| 2019/04/15 | 24.8474528 | 120.9244972 | 1 | 23.2 | 31 | 20.78 | 1.2 | ±0.1 | - |
| 2019/04/16 | 23.9719694 | 120.3238806 | 1 | 23 | 31 | 20.84 | 1.1 | ±0.2 | - |
| 2019/04/16 | 23.4530389 | 120.1381556 | 1 | 24.7 | 31.5 | 20.72 | 1.3 | ±0.2 | - |
| 2019/04/19 | 22.6175333 | 120.2681806 | 1 | 29.3 | 30.4 | 18.48 | 1.3 | ±0.2 | - |
| 2019/04/19 | 24.5819778 | 121.86835 | 1 | 25.3 | 31.4 | 20.47 | 1.2 | ±0.2 | - |
| 2019/04/19 | 25.1445778 | 121.7914778 | 1 | 26.4 | 33.1 | 21.39 | 1.1 | ±0.2 | - |
| 2019/05/01 | 21.8000056 | 120.7000028 | 1 | 28.5 | 33.5 | 21.02 | 1.0 | ±0.3 | - |
| 2019/05/01 | 21.96 | 120.59 | 1 | 28.5 | 33.7 | 21.16 | 1.3 | ±0.3 | - |
| 2019/05/01 | 21.8599972 | 120.93 | 1 | 28.5 | 33.7 | 21.16 | 1.3 | ±0.3 | - |
| 2019/05/30 | 20.4833333 | 116.6833333 | 1 | 29.9 | 32.9 | 20.11 | 1.1 | ±0.2 | - |
| 2019/05/31 | 20.5833333 | 116.75 | 1 | 29.9 | 32.3 | 19.67 | 1.4 | ±0.2 | - |
| 2019/06/01 | 20.8833333 | 116.8666667 | 1 | 30.1 | 33.1 | 20.19 | 0.9 | ±0.2 | - |
| 2019/06/18 | 23.9 | 119.5833333 | 1 | 24 | 33.5 | 22.41 | 1.4 | ±0.2 | - |
| 2019/06/18 | 23.7166667 | 119.3166667 | 1 | 24 | 33.2 | 22.19 | 1.3 | ±0.2 | - |
| 2019/06/18 | 23.3333333 | 119.6666667 | 1 | 24 | 33.5 | 22.41 | 1.7 | ±0.1 | - |
| 2019/06/25 | 25.4725 | 121.5963889 | 1 | 30.7 | 29.1 | 17.07 | 0.9 | ±0.2 | - |
| 2019/06/25 | 25.3519444 | 121.7716667 | 1 | 29.2 | 30.1 | 18.30 | 1.1 | ±0.2 | - |
| 2019/06/25 | 25.3455556 | 121.8205556 | 1 | 30.5 | 29 | 17.06 | 1.4 | ±0 | - |
| 2019/07/01 | 23.9811667 | 121.6243611 | 1 | 31.3 | 28.1 | 16.14 | 0.9 | ±0.2 | - |
| 2019/07/02 | 23.1594722 | 121.4030278 | 1 | 29.3 | 32.1 | 19.73 | 1.4 | ±0.2 | - |
| 2019/07/02 | 22.7908056 | 121.19225 | 1 | 29.1 | 33 | 20.45 | 1.1 | ±0.1 | - |
| 2019/06/30 | 10.5833333 | 114.4 | 1 | 30.1 | 32.8 | 19.97 | 1.0 | ±0.2 | - |
| 2019/06/30 | 10.6666667 | 114.65 | 1 | 30 | 33 | 20.15 | 1.2 | ±0.2 | - |
| 2019/06/30 | 10.7 | 114.8333333 | 1 | 30.2 | 33 | 20.08 | 1.4 | ±0.2 | - |
| 2019/07/16 | 24.8474528 | 120.9244972 | 1 | 31.2 | 31 | 18.29 | 1.5 | ±0.2 | - |
| 2019/07/17 | 23.9719694 | 120.3238806 | 1 | 31.3 | 30.1 | 17.60 | 1.7 | ±0.2 | - |
| 2019/07/17 | 23.4530389 | 120.1381556 | 1 | 33.2 | 30.1 | 16.94 | 1.4 | ±0.1 | - |
| 2019/07/23 | 24.5819778 | 121.86835 | 1 | 30.3 | 32.5 | 19.68 | 1.1 | ±0.2 | - |
| 2019/07/23 | 25.1445778 | 121.7914778 | 1 | 29.5 | 31.8 | 19.44 | 1.2 | ±0.2 | - |
| 2019/07/31 | 22.6175333 | 120.2681806 | 1 | 31.8 | 31.1 | 18.15 | 1.3 | ±0.2 | - |
| 2019/07/16 | 26.04 | 119.87 | 1 | 27.1 | 30.1 | 18.96 | 1.1 | ±0.1 | - |
| 2019/07/16 | 26.06 | 120 | 1 | 27.2 | 30.2 | 19.01 | 1.3 | ±0.2 | - |
| 2019/07/16 | 26.14 | 120.08 | 1 | 26.9 | 30.2 | 19.10 | 1.1 | ±0.2 | - |
| 2019/08/10 | 26.4666667 | 120.6166667 | 1 | 30.7 | 32.8 | 19.77 | 1.3 | ±0.2 | - |
| 2019/08/10 | 26.2833333 | 120.6166667 | 1 | 30.5 | 32.8 | 19.84 | 1.3 | ±0.2 | - |
| 2019/08/10 | 26.3 | 120.4 | 1 | 30.6 | 32.6 | 19.66 | 1.5 | ±0.2 | - |
| 2019/08/21 | 21.9 | 121.7 | 1 | 29.8 | 32.1 | 19.56 | 1.2 | ±0.1 | - |
| 2019/08/21 | 21.9 | 121.5 | 1 | 29.7 | 32.1 | 19.59 | 1.0 | ±0.2 | - |
| 2019/08/21 | 22 | 121.7 | 1 | 29.9 | 32.2 | 19.60 | 1.3 | ±0.1 | - |
| 2019/08/09 | 24.4 | 118.5 | 1 | 30 | 31 | 18.69 | 1.3 | ±0.1 | - |
| 2019/08/11 | 24.37 | 118.45 | 1 | 32 | 31.2 | 18.15 | 1.4 | ±0.2 | - |
| 2019/08/15 | 24.35 | 118.32 | 1 | 31 | 31.2 | 18.50 | 1.4 | ±0.2 | - |
| 2019/10/01 | 24.8474528 | 120.9244972 | 1 | 26.4 | 21.8 | 13.07 | 0.7 | ±0.1 | - |
| 2019/10/02 | 23.9719694 | 120.3238806 | 1 | 30.4 | 31.7 | 19.07 | 0.9 | ±0.2 | - |
| 2019/10/02 | 23.4530389 | 120.1381556 | 1 | 28.4 | 30.4 | 18.77 | 1.1 | ±0.2 | - |
| 2019/10/07 | 23.9811667 | 121.6243611 | 1 | 29 | 29 | 17.56 | 1.2 | ±0.2 | - |
| 2019/10/08 | 23.1594722 | 121.4030278 | 1 | 28.6 | 32.3 | 20.10 | 1.3 | ±0.2 | - |
| 2019/10/08 | 22.7908056 | 121.19225 | 1 | 28.7 | 32.4 | 20.14 | 1.4 | ±0.2 | - |
| 2019/10/15 | 22.6175333 | 120.2681806 | 1 | 31 | 31.1 | 18.43 | 1.4 | ±0.2 | - |
| 2019/10/15 | 24.5819778 | 121.86835 | 1 | 26.5 | 24.4 | 14.95 | 1.1 | ±0.2 | - |
| 2019/10/18 | 25.1445778 | 121.7914778 | 1 | 28.7 | 32.6 | 20.29 | 1.4 | ±0.1 | - |
| 2019/10/29 | 25.4725 | 121.5963889 | 1 | 23.6 | 28.7 | 18.96 | 1.7 | ±0.2 | - |
| 2019/10/29 | 25.3519444 | 121.7716667 | 1 | 23.6 | 28.5 | 18.82 | 1.0 | ±0.2 | - |
| 2019/10/29 | 25.3455556 | 121.8205556 | 1 | 23.5 | 28.8 | 19.07 | 1.3 | ±0.2 | - |
| 2019/10/25 | 25.17565 | 121.7636 | 3 | 24.3 | 33.8 | 22.55 | 1.4 | ±0.2 | - |
| 2019/10/25 | 25.17565 | 121.7636 | 30 | 24.2 | 33.8 | 22.70 | 1.5 | ±0.3 | - |
| 2019/11/13 | 21.89775 | 120.7493056 | 1 | 26.21 | 34.62 | 22.57 | 1.2 | ±0.2 | - |
| 2019/11/13 | 21.9056389 | 120.8140833 | 1 | 25.87 | 34.4759 | 22.57 | 1.5 | ±0.2 | - |
| 2019/11/13 | 21.95525 | 120.7624444 | 1 | 26.55 | 34.84 | 22.63 | 0.9 | ±0.2 | - |
| 2019/11/23 | 22.3669 | 120.2775 | 5 | 26.459 | 33.8987 | 21.98 | 1.0 | ±0.1 | - |
| 2019/11/23 | 22.3669 | 120.2775 | 200 | 15.6563 | 34.5741 | 26.26 | 1.4 | ±0.2 | - |
| 2019/11/24 | 22.3669 | 120.2775 | 400 | 9.2739 | 34.401 | 28.28 | 1.0 | ±0.2 | - |
